# Dual Probabilistic Linguistic Full Consistency Additive Ratio Assessment Model for Medical Equipment Supplier Selection

**DOI:** 10.1007/s40815-023-01526-w

**Published:** 2023-05-08

**Authors:** Arunodaya Raj Mishra, Pratibha Rani, Ibrahim M. Hezam, Muhammet Deveci

**Affiliations:** 1 Department of Mathematics, Government College Raigaon, Satna, Madhya Pradesh 485441 India; 2 Department of Engineering Mathematics, Koneru Lakshmaiah Education Foundation, Guntur, Andhra Pradesh 522302 India; 3 Department of Statistics & Operations Research, College of Sciences, King Saud University, Riyadh, Saudi Arabia; 4 Department of Industrial Engineering, Turkish Naval Academy, National Defence University, Tuzla, 34940 Istanbul, Turkey; 5 The Bartlett School of Sustainable Construction, University College London, London, WC1E 6BT UK

**Keywords:** Linguistic term sets, Probabilistic linguistic term set, DPLTSs, Power operator, Dombi operators, FUCOM, ARAS, Multi-criteria decision analysis

## Abstract

In comparison to probabilistic linguistic term sets (PLTSs), dual probabilistic linguistic term sets (DPLTSs) reveal the complexity and uncertainty of this realistic world more accurately. The power average operator can mitigate the consequences of some decision-makers’ strong biases while evaluating data. Additionally, the Dombi operators during the phase of aggregation are very adaptable with general parameter. Inspired by these topics, we propose some dual probabilistic linguistic Dombi power aggregation operators to aggregate the decision-making expert’s preferences and various elegant properties of these proposed dual probabilistic linguistic (DPL)-aggregating operators. Further, we establish an integrated method with the combination of proposed DPL-aggregation operators, full consistency method (FUCOM) and additive ratio assessment (ARAS) model called the “DPL-FUCOM-ARAS” methodology for solving multi-criteria decision analysis problems under dual probabilistic linguistic environment. In this methodology, the FUCOM is applied to determine criteria weight, while the ARAS model is presented to prioritize the options from dual probabilistic linguistic perspective. We present a case study on the selection of a medical equipment supplier to highlight the applicability and show the usefulness of the proposed DPL-FUCOM-ARAS methodology. Further, we discuss the sensitivity investigation and comparative discussion to check the stability and robustness of the proposed DPL-FUCOM-ARAS methodology.

**Supplementary Information:**

The online version contains supplementary material available at 10.1007/s40815-023-01526-w.

## Introduction

With the increasing complexity of decision-making problems and irrationality of human behavior, “decision-making experts (DMEs)” find it difficult to provide decision information as clear values. Despite the inaccuracy or uncertainty of the evaluation information collected from experts, DMEs are unable to articulate their preferences by delivering a definite, crisp number. Herrera and Martinez [1] provided “linguistic term sets (LTSs)” to easily depict qualitative evaluation information. However, there may be hesitancy among numerous “linguistic terms (LTs)” when DMEs attempt to accurately characterize their views with only one LT. For instance, a DME may use any LT occurring in the LTS, wherein *Y* = {*y*_0_: very bad, *y*_1_: bad, *y*_2_: quite bad, *y*_3_: fair, *y*_4_: quite good, *y*_5_: good, *y*_6_: very good}, to represent the performance of a medical-claim policy. The policy can be rated as “5” if a DME believes it to be good. However, in a real “multi-criteria decision analysis (MCDA)” dilemma, DMEs may arrive at various performance ratings for the same problem because of the disparity between their cognitive abilities and the difficulty of the MCDA environment. To conquer this issue, Rodriguez et al. [2] initiated the idea of a “hesitant fuzzy linguistic term set (HFLTS)”, which enables DMEs to express their prioritization for alternatives with diverse potential LTs, in order to get over the drawback of the LV. But in order to emphasize the scenario of reluctance, HFLTSs give equal weights or relevance to all potential assessment values. It is well recognized that while DMEs may have varying degrees of preference with respect to a number of potential LTs, the weights of linguistic judgments cannot be neglected in real MCDA problems. In contrast to reality, the HFLTS gives all of its items a same weight. Due to the fact that DMEs offer a variety of LTs, the HFLTS’s objects should be relevant in variable degrees. For instance, the evaluation information includes probabilistic data in addition to LTs if a DME is 70% confident that a medical-claim policy is good and 30% confident that the policy is fair. Pang et al. [3] created “probabilistic linguistic term set (PLTS)” to portray the LTS with certain probable linguistic term and related probabilistic information in order to circumvent this issue. PLTSs provide more perspective to articulate the preferences with the associated probability data for each LT. With the PLTS, Liao et al. [4] created a programming framework for evaluating hospitals. In the context of PLTSs, Zhang and Xing [5] introduced a decision support system to tackle with green supplier assessment. Kobina et al. [6] presented several “aggregation operators (AOs)” for PLTSs and further applied to solve MCDA problem. Additionally, Liu and Teng [7] created a brand-new “TOmada de Decisao Interativa Multicriterio (TODIM)” technique based on PLTSs to assess online goods using user-generated content.

Han et al. [8] proposed a PLTS-based three-way MCDA model and its application in air quality index assessment. Teng et al. [9] gave an improved power AOs for PLTSs. Zhou et al. [10] suggested a hybrid MCDA framework by uniting the “best–worst method (BWM)” and TODIM method under PLTS context. Han and Zhan [11] designed a three-way MCDA-based consensus model on PLTSs environment. As a result, the PLTS has recently received increased attention [12–15]. Xie et al. [16] extended the PLTS to dual PLTS (DPLTS), which can express the decision analysis data with combination of the “membership grade (MG)” and “non-membership grade (NG)”. They suggested the use of arithmetic AO to address the MCDA issues in the context of DPLTSs. An integrated DPLTS-based MCDA model with preference relations and information fusion has been developed by Xie et al. [17]. A DPL methodology with generalised Dombi and Bonferroni mean operators was presented by Saha et al. [18]. In order to deal with the difficulty of choosing a biomass feedstock, Saha et al. [19] addressed “measurement alternatives and ranking based on compromise solution (MARCOS)” employing the generalised Dombi operator in a dual probabilistic linguistic environment.

There are several concerns for dealing with DPL data are identified from thorough analysis discussed as.

1. The approach outlined by Xie et al. [16] cannot completely rule out the influence of highly skewed assessment criterion values from various biased DMEs with a range of preferred views.
2. It is obvious that not all criteria are taken into account equally in some situations that are grounded in reality. For the position of sales manager, for instance, a candidate’s working history is given preference over their educational background and age. As a result, preference must be carefully considered while choosing the right criteria weight. The authors of the current technique [16] set the attribute weights for the final aggregation procedure randomly, which has an impact on the final ranking order. Additionally, the earlier DPLTS studies [16–19] are unable to address a scenario in which the preferred association among the attribute is only identified for the purpose of determining the weights of criteria.
3. Arithmetic AOs [16] are used only for aggregating the information, however, a more flexible operator that can be modified in accordance with the actual decision needs is still urgently needed.
4. Till now, no one has used any of the well-known ranking techniques, such as “technique for Order preference by similarity to ideal solution (TOPSIS)”, “complex proportional assessment (COPRAS)”, TODIM, “multi attributive ideal-real comparative analysis (MAIRCA)” or “additive ratio assessment (ARAS)” in order to determine the ranking preferences for alternatives. Therefore, prioritizing alternatives by extending common ranking techniques in a DPLTS scenario is still a difficult problem.

In order to address the aforementioned problems, the key contributions of the paper are given by

1. This paper presents some operations for DPLTSs by making use of Dombi and Power operations [20].
2. To lessen the effects of absurd information from some biased DMEs during the MCDA practice, the Dombi power weighted averaging and geometric AOs are presented on DPLTSs context. These AOs have a parameter called “*Q*” that allows for change in accordance with the actual decision-making requirements.
3. The criteria weights are calculated using “full consistency method (FUCOM)” [21]. However, FUCOM provided the lesser deviations of accomplished degree of the criterion from the most desirable values compared to the “analytic hierarchy process (AHP)”, BWM and another subjective weighting processes [21].
4. Allowing to Zavadskas and Turskis [22], the ARAS model is more logical as a result of the combining of reference point sorting and ratio approach results. By leveraging the aforementioned power weighted AOs and the advantages of the ARAS method, a unique FUCOM-ARAS model is provided in this study to address “multi-criteria group decision analysis (MCGDA)” difficulties with dual probabilistic linguistic (DPL) data.
5. A case study of selecting a “medical equipment supplier (MES)” is discussed in the context of a DPL setting to clarify the applicability and value of the suggested approach. To validate the findings of the suggested framework, a sensitivity investigation is presented. In order to prove that the introduced model is superior, a comparison is presented last.

The rest part is organized as we provide thorough literature survey on Dombi operations and the ARAS approach in Sect. 2 of this article. In Sect. 3, we provide some key ideas about DPLTSs, Dombi operations, and power average operator. We create Dombi operating regulations for DPL elements (DPLEs) in Sect. 4 and investigate their characteristics in relation to DPL Dombi power AOs like “dual probabilistic linguistic Dombi power weighted averaging aggregation (DPLDPWAA)” and “dual probabilistic linguistic Dombi power weighted geometric aggregation (DPLDPWGA)” operators. Section 5 discusses a novel FUCOM-ARAS methodology that makes use of the suggested power AOs and expresses the criteria values as DPLEs. A case study of selecting a MES is used to explain the proposed technique in Sect. 6. The discussion of the results is the only focus of Sect. 7. This includes how the parameter affects the rankings. A comparative discussion is used to demonstrate the advantage of the created approach at the conclusion of this section. A few conclusions on the study are discussed in Sect. 8.

## Related Works

This section shows the comprehensive literatures related to the current work.

### Dombi Operators

The Dombi operator, introduced by Dombi [20], is unusual since it has high parameter flexibility and can tell by the parameter’s sign whether it is conjunctive or disjunctive. He [23] looked at the Dombi operations in a tentative, hazy environment when assessing typhoon disasters. In a Pythagorean fuzzy environment, Akram et al. [24] gave a set of Dombi AOs with important properties as idempotency, monotonicity, boundedness, reducibility, and commutativity. Ashraf et al. [25] developed numerous Dombi AOs under the framework of “spherical fuzzy set (SFS)”, including the geometric, hybrid, Dombi weighted averaging, and discussed their features in detail. Kurama [26] used some Dombi AOs through aggregation of similarities using the classifier. Karaaslan and Dawood [27] proposed a series of Dombi weighted AOs for T-spherical fuzzy set. Saha et al. [18] developed a set AOs on HFSs by combining the advantages of Archimedean and Dombi AOs, and utilized these AOs to deal with personnel selection problem under HFSs. Liu et al. [28] presented some general as well as flexible AOs combining the benefits of Dombi and Archimedean AOs to solve MCDA problems with the HFSs settings. Saha et al. [19] discussed the MARCOS using generalized Dombi operator under dual probabilistic linguistic setting to deal with biomass feedstock selection problem. Kavitha et al. [29] utilized the hesitant q-rung orthopair fuzzy Dombi AOs for feature selection. However, there is no work about the unification of PAO and Dombi AOs with DPL setting.

### ARAS Method

The ARAS model considers a utility degree determining the relative efficiency of a feasible option is directly proportional to the relative degree of options and weight of key criteria. Zavadskas and Turkis [22] pioneered the ARAS model, indicating that the events of this intricate world may be implicit using easy relative comparisons. ARAS makes use of the concept of an optimality degree in order to achieve prioritization. The most important benefits of ARAS include: (1) direct and proportional relationship with attribute weights [30], (2) having the ability to solve complicated problems [31], (3) involving some simple and direct steps for the assessment of a number of options or choices based on their performance in comparison with the chosen evaluation criteria that obtained suitable, sensible, and comparatively accurate results [22]. In recent years, this approach has been elaborated in various uncertain fields [32–38]. Karimi and Nikkhah-Farkhani [39] assessed the performance of workers in an academic center of education through augmented ARAS method. Jovčić et al. [40] studied an integrated picture fuzzy ARAS methodology for freight distribution assessment. Gul [41] generalized the ARAS method from Fermatean fuzzy perspective with an application in COVID-19 testing lab selection. The classical ARAS method has extended under “q-rung orthopair fuzzy sets (q-ROFSs)” setting by Mishra and Rani [37]. In that study, the authors have proven the applicability of the developed model through comparative and sensitivity analyses. Liu and Xu [42] presented the literature survey on ARAS method with its applications and challenges. Karagöz [43] incorporated ARAS with “interval type-2 fuzzy sets (IT2FSs)” for the evaluation of recycling facility locations from SD context. Rani et al. [44] presented a Fermatean fuzzy information-ARAS method with the application in a food waste treatment technology selection problem. Liu and Mishra [45] aimed to rank and evaluate the challenges to implement the green internet of things (G-IoT) towards the sustainable development achievements (SDA) using an integrated approach with the SWARA and the ARAS under Pythagorean fuzzy sets (PFSs). Hu et al. [46] evaluated and ranked the IoT risks for *s*upply chain management (SCM) by utilizing the “Stepwise Weight Assessment Ratio Analysis (SWARA)” and the ARAS tools under q-ROFSs. Dahooie et al. [47] combined the classical approach with “Data envelopment analysis (DEA)” and fuzzy information. A biomass crop selection problem has evaluated through a hybrid Pythagorean fuzzy ARAS method [48]. Mentes and Akyildiz [49] suggested a MCDA model using AOs and ARAS tool and used for criticality analysis. Yet, there is no study regarding the hybrid ARAS methodology with FUCOM and DPL information.

## Preliminaries

### Fundamental Definitions

#### Definition 3.1

[50] If $k$ is a natural number and $l_{\beta }$ denotes a linguistic variable, then $T = \{ l_{\beta } :\beta = - k,\ldots,k\}$ denotes a LTS provided as follows:

1. neg($$l_{\beta }$$) = $$l_{\delta },$$ where $$\beta + \delta = 2k$$.
2. $$l_{\beta } \le l_{\delta }$$ if $$\beta \le \delta$$.

Gou et al. [51] defined two mappings $$L:[ - k,k] \to [0,1]$$ and $$L^{ - 1} :[0,1] \to [ - k,k][0,1]$$$$\to [ - k,k]$$ and is given by

$$\begin{gathered} L(l_{\beta } ) = \frac{\beta }{2k} + \frac{1}{2}\,\,\,(\beta = - k,\ldots.,k) \hfill \\ L^{ - 1} (\delta ) = l_{(2\delta - 1)k} \,\,(\delta \in [0,1]) \hfill \\ \end{gathered}$$

#### Definition 3.2

[16] If $T = \{ l_{\beta } :\beta = - k,\ldots,k\}$ is a LTS, then a DPLTS $H_{l} (\Im )$ on $U = \{ y:y \in U\}$ is expressed as

$$H_{l} (\Im ) = \{ < y,\mu_{l} (\Im )(y),\gamma_{l} (\Im )(y) > :y \in U\},$$

where $\mu_{l} (\Im )(y) = \{ l_{{\varphi^{(c)} }} (\Im^{(c)} ):l_{{\varphi^{(c)} }} \in T,\Im^{(c)} \ge 0,\sum\limits_{c}^{{}} {\Im^{(c)} \le 1} \,\}$ &$\gamma_{l} (\Im )(y) = \{ l_{{\eta^{(d)} }} (\Im^{(d)} ):l_{{\eta^{(d)} }} \in T,\Im^{(d)} \ge 0,\sum\nolimits_{d}^{{}} {\Im^{(d)} \le 1} \,\}$ and the associated probabilities of $l_{{\varphi^{(c)} }}$ and $l_{{\eta^{(d)} }}$ are, respectively, $\Im^{(c)}$ and $\Im^{(d)}$.

If $H_{l} (\Im )$ is singleton, then we call it a “DPL element (DPLE)” and express it by $H_{l} (\Im ) = < \{ l_{{\varphi^{(c)} }} (\Im^{(c)} )\} ,\{ l_{{\eta^{(d)} }} (\Im^{(d)} )\} >$.

#### Definition 3.3

[16] Let $H_{l}^{m} (\Im ) = < \{ l_{{\varphi^{m(c)} }} (\Im^{m(c)} )\} ,\{ l_{{\eta^{m(d)} }} (\Im^{m(d)} )\} > \,(m = 1,2)$ be two DPLEs. For sake of simplicity, let us take, $L_{\varphi }^{m} = L(l_{{\varphi^{m(c)} }} )$ and $L_{\eta }^{m} = L(l_{{\eta^{m(d)} }} )\,(m = 1,2)$. Then

(i) $$Neg(H_{l}^{1} (\Im )) = < \{ l_{{\eta^{m(d)} }} (\Im^{m(d)} )\} ,\{ l_{{\varphi^{m(c)} }} (\Im^{m(c)} )\} >$$
(ii) $$H_{l}^{1} (\Im ) \oplus H_{l}^{2} (\Im ) = \left\langle {\left\{ {L^{ - 1} \left( {L_{\varphi }^{1} + L_{\varphi }^{2} - L_{\varphi }^{1} L_{\varphi }^{2} } \right)\,(\Im^{1(c)} \Im^{2(c)} )} \right\},\,L^{ - 1} \left( {L_{\eta }^{1} \,L_{\eta }^{2} } \right)\,(\Im^{1(d)} \Im^{2(d)} )} \right\rangle$$
(iii) $$H_{l}^{1} (\Im ) \otimes H_{l}^{2} (\Im ) = \left\langle {\left\{ {L^{ - 1} \left( {L_{\varphi }^{1} L_{\varphi }^{2} } \right)\,(\Im^{1(c)} \Im^{2(c)} )} \right\},\,L^{ - 1} \left( {L_{\eta }^{1} \, + L_{\eta }^{2} - L_{\eta }^{1} \,L_{\eta }^{2} } \right)\,(\Im^{1(d)} \Im^{2(d)} )} \right\rangle$$
(iv) $$\lambda H_{l}^{1} (\Im ) = \left\langle {\left\{ {L^{ - 1} \left( {1 - (1 - L_{\varphi }^{1} )^{\lambda } } \right)\,(\Im^{1(c)} )} \right\},\,L^{ - 1} \left( {(L_{\eta }^{1} )^{\lambda } } \right)\,(\Im^{1(d)} )} \right\rangle$$
(v) $$(H_{l}^{1} (\Im ))^{\lambda } = \left\langle {L^{ - 1} \left( {(L_{\varphi }^{1} )^{\lambda } } \right)\,(\Im^{1(c)} ),\left\{ {L^{ - 1} \left( {1 - (1 - L_{\eta }^{1} )^{\lambda } } \right)\,(\Im^{1(d)} )} \right\}} \right\rangle$$

#### Theorem 3.1

[16] For any $\lambda ,\lambda_{1} ,\lambda_{2} > 0$, we have

1. $$H_{l}^{1} (\Im ) \oplus H_{l}^{2} (\Im ) = H_{l}^{2} (\Im ) \oplus H_{l}^{1} (\Im )$$
2. $$H_{l}^{1} (\Im ) \otimes H_{l}^{2} (\Im ) = H_{l}^{2} (\Im ) \otimes H_{l}^{1} (\Im )$$
3. $$\lambda (H_{l}^{1} (\Im ) \oplus H_{l}^{2} (\Im )) = (\lambda H_{l}^{1} (\Im )) \oplus (\lambda H_{l}^{2} (\Im ))$$
4. $$(H_{l}^{1} (\Im ) \otimes H_{l}^{2} (\Im ))^{\lambda } = (H_{l}^{1} (\Im ))^{\lambda } \otimes (H_{l}^{2} (\Im ))^{\lambda }$$
5. $$(\lambda_{1} + \lambda_{2} )H_{l}^{1} (\Im ) = (\lambda_{1} H_{l}^{1} (\Im )) \oplus (\lambda_{2} H_{l}^{1} (\Im ))$$
6. $$(H_{l}^{1} (\Im ))^{{\lambda_{1} + \lambda_{2} }} = (H_{l}^{1} (\Im ))^{{\lambda_{1} }} \otimes (H_{l}^{1} (\Im ))^{{\lambda_{2} }}$$

The score and accuracy values of a DPLE were defined by Xie et al. [16]. But the mathematical structures of the calculations they utilized are extremely sophisticated. The procedure is described below to simplify those.

#### Definition 3.4

Let $H_{l} (\Im ) = < \{ l_{{\varphi^{(c)} }} (\Im^{(c)} )\} ,\{ l_{{\eta^{(d)} }} (\Im^{(d)} )\} >$ be a DPLE. Then the score value of $H_{l} (\Im )$ is given by

1 $$S(H_{l} (\Im )) = \sum\limits_{c}^{{}} {L(l_{{\varphi^{(c)} }} )(\Im^{(c)} )} - \sum\limits_{d}^{{}} {L(l_{{\eta^{(d)} }} )(\Im^{(d)} )}.$$

The better the DPLEs, the higher the value of $S(H_{l} (\Im ))$. However, using the same score values to compare the DPLEs are insufficient. In the following, the idea of accuracy value is introduced:

#### Definition 3.5

Let $H_{l} (\Im ) = < \{ l_{{\varphi^{(c)} }} (\Im^{(c)} )\} ,\{ l_{{\eta^{(d)} }} (\Im^{(d)} )\} >$ be a DPLE. Then the accuracy value of $H_{l} (\Im )$ is given by

2 $$S(H_{l} (\Im )) = \sum\limits_{c}^{{}} {L(l_{{\varphi^{(c)} }} )(\Im^{(c)} )} + \sum\limits_{d}^{{}} {L(l_{{\eta^{(d)} }} )(\Im^{(d)} )}.$$

#### Definition 3.6

[16] Let $H_{l}^{m} (\Im ) = < \{ l_{{\varphi^{m(c)} }} (\Im^{m(c)} )\} ,\{ l_{{\eta^{m(d)} }} (\Im^{m(d)} )\} > \,(m = 1,2)$ be two DPLEs. Then, an ordering scheme for DPLEs is given by

A. If $$S(H_{l}^{1} (\Im )) > S(H_{l}^{2} (\Im ))$$, then $$H_{l}^{1} (\Im ) \succ H_{l}^{2} (\Im ),$$
B. If $$S(H_{l}^{1} (\Im )) = S(H_{l}^{2} (\Im ))$$, then
  - If $$A(H_{l}^{1} (\Im )) > A(H_{l}^{2} (\Im ))$$, then $$H_{l}^{1} (\Im ) \succ H_{l}^{2} (\Im ),$$
  - If $$A(H_{l}^{1} (\Im )) = A(H_{l}^{2} (\Im ))$$, then $$H_{l}^{1} (\Im ) = H_{l}^{2} (\Im ).$$

### Dombi Operations

Dombi operations [20] are the t-norm and t-conorm operations that were created in 1982. They are detailed below:

#### Definition 3.7

[20] If 0 ≤ *p*, *q* ≤ 1, then the Dombi *t*-norm and Dombi *t*-conorm are presented as:

$$Dom(p,q) = \left( {1 + \left\{ {\left( {(1 - p)/p} \right)^{Q} + \left( {(1 - q)/q} \right)^{Q} } \right\}^{\frac{1}{Q}} } \right)^{ - 1} ,\,Dom^{c} (p,q) = 1 - \left( {1 + \left\{ {\left( {q/(1 - q)} \right)^{Q} + \left( {p/(1 - p)} \right)^{Q} } \right\}^{\frac{1}{Q}} } \right)^{ - 1} (Q \ge 1).$$

### Power Average Operator (PAO)

#### Definition 3.8

[52] If $t_{1} ,t_{2} ,\,\ldots,t_{n}$ are crisp values, then the power average operator (PAO) operator is expressed as

3 $$PA(t_{1} ,t_{2} ,\,\ldots,t_{r} ) = \frac{{\sum\nolimits_{j = 1}^{r} {\left( {1 + \rho (t_{j} )} \right)t_{j} } }}{{\sum\nolimits_{j = 1}^{r} {\left( {1 + \rho (t_{j} )} \right)} }},\;{\text{where}}\;\rho \left( {t_{j} } \right) = \sum\limits_{i = 1,j \ne i}^{r} {ST\left( {t_{i} ,t_{j} } \right)} .$$

Here, $ST(t_{i} ,t_{j} )$ signifies the support for $t_{i}$ from $t_{j}$ and holds subsequent axioms as

(i) $$0 \le ST(t_{i} ,t_{j} ) \le 1$$
(ii) $$ST(t_{i} ,t_{j} ) = ST(t_{j} ,t_{i} )$$
(iii) ($ST(t_{i} ,t_{j} ) \ge ST(t_{s} ,t_{u} )$ provided $\left| {t_{i} - t_{j} } \right| < \left| {t_{s} - t_{u} } \right|,$ where $i,j,s,u$ are all natural numbers.

## Dombi Power Weighed Aggregation Operators

In certain cases the probabilities and the LTs in the DPLEs $H_{l}^{1} (\Im )$ and $H_{l}^{2} (\Im )$ are different. In the process of aggregating information, multiplying with probabilities and their consequent linguistic ratings may result in irrational outcomes. We use the probability adjustment approach, whose process is illustrated in the example below, to deal with this problem.

### Example 1

**Fig. 1:**
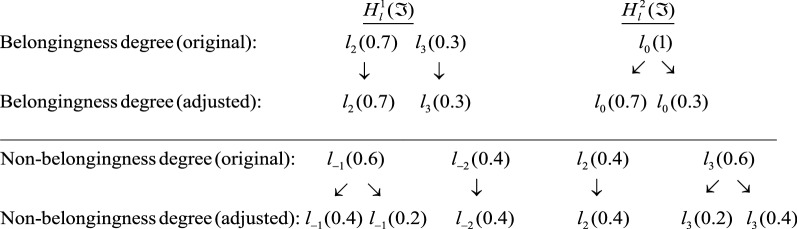
Adjustments of DPLEs

[19] Let $T = \{ l_{ - 3} ,l_{ - 2} ,l_{ - 1} ,l_{0} ,l_{1} ,l_{2} ,l_{3} \}$ be a discrete LTS. Take two DPLEs $H_{l}^{1} (\Im ) = < \{ l_{2} (0.7),l_{3} (0.3)\} ,\,\{ l_{ - 1} (0.6),l_{ - 2} (0.4)\} >$ and $H_{l}^{2} (\Im ) = < \{ l_{0} (1)\} ,\,\{ l_{2} (0.4),l_{3} (0.6)\} > .$ Then their corresponding adjusted DPLEs are: $\tilde{H}_{l}^{1} (\Im ) = < \{ l_{2} (0.7),l_{3} (0.3)\} ,\,\{ l_{ - 1} (0.4),l_{ - 1} (0.2),l_{ - 2} (0.4)\} >$ and $\tilde{H}_{l}^{2} (\Im ) = < \{ l_{0} (0.7),l_{0} (0.3)\} ,\,\{ l_{2} (0.4),l_{3} (0.2),l_{3} (0.4)\} >$. The technique of adjustment is (see Fig. 1).

### Definition 3.9

For the adjusted DPLEs $H_{l}^{m} (\Im ) = \,\left\langle {\{ l_{{\varphi^{m(c)} }} (\Im^{m(c)} )\} ,\{ l_{{\eta^{m(d)} }} (\Im^{m(d)} )\} } \right\rangle ,m = 1,2,$ the Dombi operations are defined by

- 4 $$\tilde{H}_{l}^{1} (\Im )\tilde{ \oplus }\tilde{H}_{l}^{2} (\Im ) = \left\langle {\left\{ {L^{ - 1} \left( {1 - \left( {1 + \left\{ {\sum\limits_{j = 1}^{2} {\left( {\frac{{L_{\varphi }^{j} }}{{1 - L_{\varphi }^{j} }}} \right)^{Q} } } \right\}^{\frac{1}{Q}} } \right)^{ - 1} } \right)\,(\Im^{(c)} )} \right\},\,\left\{ {L^{ - 1} \left( {\left( {1 + \left\{ {\sum\limits_{j = 1}^{2} {\left( {\frac{{1 - L_{\eta }^{j} }}{{L_{\eta }^{j} }}} \right)^{Q} } } \right\}^{\frac{1}{Q}} } \right)^{ - 1} } \right)\,(\Im^{(d)} )} \right\}} \right\rangle,$$
- 5 $$\tilde{H}_{l}^{1} (\Im )\tilde{ \otimes }\tilde{H}_{l}^{2} (\Im ) = \left\langle {\left\{ {L^{ - 1} \left( {\left( {1 + \left\{ {\sum\limits_{j = 1}^{2} {\left( {\frac{{1 - L_{\varphi }^{j} }}{{L_{\varphi }^{j} }}} \right)^{Q} } } \right\}^{\frac{1}{Q}} } \right)^{ - 1} } \right)\,(\Im^{(c)} )} \right\},\,\left\{ {L^{ - 1} \left( {1 - \left( {1 + \left\{ {\sum\limits_{j = 1}^{2} {\left( {\frac{{L_{\eta }^{j} }}{{1 - L_{\eta }^{j} }}} \right)^{Q} } } \right\}^{\frac{1}{Q}} } \right)^{ - 1} } \right)\,(\Im^{(d)} )} \right\}} \right\rangle,$$
- 6 $$\lambda \tilde{H}_{l}^{1} (\Im ) = \left\langle {\left\{ {L^{ - 1} \left( {1 - \left( {1 + \left\{ {\lambda \left( {\frac{{L_{\varphi }^{1} }}{{1 - L_{\varphi }^{1} }}} \right)^{Q} } \right\}^{\frac{1}{Q}} } \right)^{ - 1} } \right)\,(\Im^{(c)} )} \right\},\,\left\{ {L^{ - 1} \left( {\left( {1 + \left\{ {\lambda \left( {\frac{{1 - L_{\eta }^{1} }}{{L_{\eta }^{1} }}} \right)^{Q} } \right\}^{\frac{1}{Q}} } \right)^{ - 1} } \right)\,(\Im^{(d)} )} \right\}} \right\rangle,$$
- 7 $$(\tilde{H}_{l}^{1} (\Im ))^{\lambda } = \left\langle {\left\{ {L^{ - 1} \left( {\left( {1 + \left\{ {\lambda \left( {\frac{{1 - L_{\varphi }^{1} }}{{L_{\varphi }^{1} }}} \right)^{Q} } \right\}^{\frac{1}{Q}} } \right)^{ - 1} } \right)\,(\Im^{(c)} )} \right\},\,\left\{ {L^{ - 1} \left( {1 - \left( {1 + \left\{ {\lambda \left( {\frac{{L_{\eta }^{1} }}{{1 - L_{\eta }^{1} }}} \right)^{Q} } \right\}^{\frac{1}{Q}} } \right)^{ - 1} } \right)\,(\Im^{(d)} )} \right\}} \right\rangle$$

### Theorem 3.2

For any $\lambda ,\lambda_{1} ,\lambda_{2} > 0$, we have

1. $$\tilde{H}_{l}^{1} (\Im )\tilde{ \oplus }\tilde{H}_{l}^{2} (\Im ) = \tilde{H}_{l}^{2} (\Im )\tilde{ \oplus }\tilde{H}_{l}^{1} (\Im )$$
2. $$\tilde{H}_{l}^{1} (\Im )\tilde{ \otimes }\tilde{H}_{l}^{2} (\Im ) = \tilde{H}_{l}^{2} (\Im )\tilde{ \otimes }\tilde{H}_{l}^{1} (\Im )$$
3. $$\lambda (\tilde{H}_{l}^{1} (\Im )\tilde{ \oplus }\tilde{H}_{l}^{2} (\Im )) = (\lambda \tilde{H}_{l}^{1} (\Im ))\tilde{ \oplus }(\lambda \tilde{H}_{l}^{2} (\Im ))$$
4. $$(\tilde{H}_{l}^{1} (\Im )\tilde{ \otimes }\tilde{H}_{l}^{2} (\Im ))^{\lambda } = (\tilde{H}_{l}^{1} (\Im ))^{\lambda } \tilde{ \otimes }(\tilde{H}_{l}^{2} (\Im ))^{\lambda }$$
5. $$(\lambda_{1} + \lambda_{2} )\tilde{H}_{l}^{1} (\Im ) = (\lambda_{1} \tilde{H}_{l}^{1} (\Im ))\tilde{ \oplus }(\lambda_{2} \tilde{H}_{l}^{1} (\Im ))$$
6. $$(\tilde{H}_{l}^{1} (\Im ))^{{\lambda_{1} + \lambda_{2} }} = (\tilde{H}_{l}^{1} (\Im ))^{{\lambda_{1} }} \tilde{ \otimes }(\tilde{H}_{l}^{1} (\Im ))^{{\lambda_{2} }}$$

### Proof

Follows from Definition 3.9.□

For rest of the paper, we assume that $\tilde{H}_{l}^{m} (\Im ) = \left\langle {\{ l_{{\varphi^{m(c)} }} (\Im^{m(c)} )\} ,\{ l_{{\eta^{m(d)} }} (\Im^{m(d)} )\} } \right\rangle ,m = 1,2,\ldots,n$ be a set of adjusted DPLEs.

### Definition 3.10

We define $DPLDPWAA$ operator as

8 $$DPLDPWAA\left( {\tilde{H}_{l}^{1} (\Im )\,,\tilde{H}_{l}^{2} (\Im )\,,\ldots,\tilde{H}_{l}^{n} (\Im )} \right)\, = \mathop {\tilde{ \oplus }}\limits_{m = 1}^{n} \left( {\varpi_{m} \tilde{H}_{l}^{m} (\Im )} \right)\,.$$

9 $${\text{Here}}\,\,\varpi_{m} = \frac{{\left( {1 + \sum\limits_{r = 1,r \ne m}^{n} {SP(\tilde{H}_{l}^{m} (\Im ),\tilde{H}_{l}^{r} (\Im ))} } \right)w_{j} }}{{\sum\limits_{m = 1}^{n} {w_{j} \left( {1 + \sum\nolimits_{r = 1,r \ne m}^{n} {SP(\tilde{H}_{l}^{m} (\Im ),\tilde{H}_{l}^{r} (\Im ))} } \right)} }},$$

where $$w_{m} ( > 0)$$ is the weight of $$\tilde{H}_{l}^{m} (\Im )$$ such that $$\sum\nolimits_{m = 1}^{n} {w_{m} } = 1$$, $$SP\left( {\tilde{H}_{l}^{m} (\Im ),\tilde{H}_{l}^{r} (\Im )} \right) = 1 - D\left( {\tilde{H}_{l}^{m} (\Im ),\tilde{H}_{l}^{r} (\Im )} \right)$$$$D\left( {\tilde{H}_{l}^{m} (\Im ),\tilde{H}_{l}^{r} (\Im )} \right)$$ being the distance between $$\tilde{H}_{l}^{m} (\Im )$$ and $$\tilde{H}_{l}^{r} (\Im )$$.

### Theorem 3.3

The aggregated value $DPLDPWAA\left( {\tilde{H}_{l}^{1} (\Im )\,,\tilde{H}_{l}^{2} (\Im )\,,\ldots,\tilde{H}_{l}^{n} (\Im )} \right)$ is again a DPLE and

$$DPLDPWAA\left( {\tilde{H}_{l}^{1} (\Im )\,,\tilde{H}_{l}^{2} (\Im )\,,\ldots,\tilde{H}_{l}^{n} (\Im )} \right)$$

10 $$= \left\langle {\left\{ {L^{ - 1} \left( {1 - \left( {1 + \left\{ {\sum\limits_{j = 1}^{2} {\varpi_{j} \left( {\frac{{L_{\varphi }^{j} }}{{1 - L_{\varphi }^{j} }}} \right)^{Q} } } \right\}^{\frac{1}{Q}} } \right)^{ - 1} } \right)\,(\Im^{(c)} )} \right\},\,\left\{ {L^{ - 1} \left( {\left( {1 + \left\{ {\sum\limits_{j = 1}^{2} {\varpi_{j} \left( {\frac{{1 - L_{\eta }^{j} }}{{L_{\eta }^{j} }}} \right)^{Q} } } \right\}^{\frac{1}{Q}} } \right)^{ - 1} } \right)\,(\Im^{(d)} )} \right\}} \right\rangle$$

### Proof

Follows from Definitions 3.9, 3.10 and Theorem 3.2.□

### Theorem 3.4 (Idempotency)

Suppose $$H_{l}^{m} \left( \Im \right) = H_{l}^{0} \left( \Im \right)\,\,\forall m\,.$$ Then, $$DPLDPWAA\left( {\tilde{H}_{l}^{1} (\Im )\,,\tilde{H}_{l}^{2} (\Im )\,,\ldots,\tilde{H}_{l}^{n} (\Im )\,} \right) = H_{l}^{0} \left( \Im \right).$$

### Theorem 3.5 (Monotonicity)

Let $H{^{\prime}_{l}}^{m} (\Im ) = \left\langle {\{ l_{{\varphi ^{{m(c)}} }}^{\prime } (\Im ^{{m(c)}} )\} ,\{ l_{{\eta ^{{m(d)}} }}^{\prime } (\Im ^{{m(d)}} )\} } \right\rangle ,{\mkern 1mu} {\mkern 1mu} m = 1,2, \ldots ,n$ be another collection of adjusted DPLEs such that ∀*m*, $l_{{\varphi^{m(c)} }} \le l^{\prime}_{{\varphi^{m(c)} }}$ and $l_{{\eta^{m(d)} }} \ge l^{\prime}_{{\eta^{m(d)} }}$. Then, $DPLDPWAA\left( {\tilde{H}_{l}^{1} (\Im )\,,\tilde{H}_{l}^{2} (\Im )\,,\ldots,\tilde{H}_{l}^{n} (\Im )\,} \right) \le DPLDPWAA\left( {\tilde{H}{^{\prime}_{l}}^{1} (\Im )\,,\tilde{H}{^{\prime}_{l}}^{2} (\Im )\,,\ldots,\tilde{H}{^{\prime}_{l}}^{n} (\Im )} \right).$

### Theorem 3.6 (Boundedness)

If $\tilde{H}_{l}^{m - } \left( \Im \right) = \left\langle {\{ \mathop {\min }\limits_{c} l_{{\varphi^{m(c)} }} (\Im^{m(c)} )\} ,\{ \mathop {\max }\limits_{d} l_{{\eta^{m(d)} }} (\Im^{m(d)} )\} } \right\rangle \,$ and $\tilde{H}_{l}^{m + } \left( \Im \right) = \left\langle {\{ \mathop {\max }\limits_{c} l_{{\varphi^{m(c)} }} (\Im^{m(c)} )\} ,\{ \mathop {\min }\limits_{d} l_{{\eta^{m(d)} }} (\Im^{m(d)} )\} } \right\rangle \,$ then

$$\tilde{H}_{l}^{m - } \left( \Im \right) \prec DPLDPWAA\left( {\tilde{H}_{l}^{1} (\Im )\,,\tilde{H}_{l}^{2} (\Im )\,,\ldots,\tilde{H}_{l}^{n} (\Im )} \right)\,\, \prec \tilde{H}_{l}^{m + } \left( \Im \right).$$

### Definition 3.11

We define the *DPLDPWGA* operator by

11 $$DPLDPWGA\left( {\tilde{H}_{l}^{1} (\Im )\,,\tilde{H}_{l}^{2} (\Im )\,,\ldots,\tilde{H}_{l}^{n} (\Im )} \right) = \mathop {\tilde{ \otimes }}\limits_{m = 1}^{n} \left( {\tilde{H}_{l}^{m} (\Im )\,} \right)^{{\varpi_{m} }} .$$

12 $${\text{Here}}\;\varpi_{m} = \frac{{\left( {1 + \sum\limits_{r = 1,r \ne m}^{n} {SP(\tilde{H}_{l}^{m} (\Im ),\tilde{H}_{l}^{r} (\Im ))} } \right)w_{j} }}{{\sum\nolimits_{m = 1}^{n} {w_{j} \left( {1 + \sum\limits_{r = 1,r \ne m}^{n} {SP(\tilde{H}_{l}^{m} (\Im ),\tilde{H}_{l}^{r} (\Im ))} } \right)} }},$$

where $w_{m} ( > 0)$ is the weight of $\tilde{H}_{l}^{m} (\Im )$ such that $\sum\nolimits_{m = 1}^{n} {w_{m} } = 1$, $SP\left( {\tilde{H}_{l}^{m} (\Im ),\tilde{H}_{l}^{r} (\Im )} \right) = 1 - D\left( {\tilde{H}_{l}^{m} (\Im ),\tilde{H}_{l}^{r} (\Im )} \right)$ and $D(\tilde{H}_{l}^{m} (\Im ),\tilde{H}_{l}^{r} (\Im ))$ being the distance between $\tilde{H}_{l}^{m} (\Im )$ and $\tilde{H}_{l}^{r} (\Im )$.

### Theorem 3.7

The aggregated value $DPLDPWGA(\tilde{H}_{l}^{1} (\Im )\,,\tilde{H}_{l}^{2} (\Im )\,,\ldots,\tilde{H}_{l}^{n} (\Im )\,)$ is again a DPLE and $DPLDPWGA\left( {\tilde{H}_{l}^{1} (\Im )\,,\tilde{H}_{l}^{2} (\Im )\,,\ldots,\tilde{H}_{l}^{n} (\Im )} \right)\,$

13 $$= \left\langle {\left\{ {L^{ - 1} \left( {\left( {1 + \left\{ {\sum\limits_{j = 1}^{2} {\varpi_{j} \left( {\frac{{1 - L_{\varphi }^{j} }}{{L_{\varphi }^{j} }}} \right)^{Q} } } \right\}^{\frac{1}{Q}} } \right)^{ - 1} } \right)\,(\Im^{(c)} )} \right\},\,\left\{ {L^{ - 1} \left( {1 - \left( {1 + \left\{ {\sum\limits_{j = 1}^{2} {\varpi_{j} \left( {\frac{{L_{\eta }^{j} }}{{1 - L_{\eta }^{j} }}} \right)^{Q} } } \right\}^{\frac{1}{Q}} } \right)^{ - 1} } \right)\,(\Im^{(d)} )} \right\}} \right\rangle$$

### Theorem 3.8 (Idempotency)

Suppose $$H_{l}^{m} (\Im ) = H_{l}^{0} (\Im )\,\,\forall m\,.$$ Then, $$DPLDPWGA\left( {\tilde{H}_{l}^{1} (\Im )\,,\tilde{H}_{l}^{2} (\Im )\,,\ldots,\tilde{H}_{l}^{n} (\Im )} \right)\, = H_{l}^{0} \left( \Im \right).$$

### Theorem 3.9 (Monotonicity)

Let $\,\tilde{H}{^{\prime}_{l}}^{m} (\Im ) = \left\langle {\{ l^{\prime}_{{\varphi^{m(c)} }} (\Im^{m(c)} )\} ,\{ l^{\prime}_{{\eta^{m(d)} }} (\Im^{m(d)} )\} } \right\rangle ,\,m = 1,2,\ldots,n$ be another collection of adjusted DPLEs such that ∀*m*, $l_{{\varphi^{m(c)} }} \le l^{\prime}_{{\varphi^{m(c)} }}$ and $l_{{\eta^{m(d)} }} \ge l^{\prime}_{{\eta^{m(d)} }}$. Then, $DPLDPWGA\left( {\tilde{H}_{l}^{1} (\Im )\,,\tilde{H}_{l}^{2} (\Im )\,,\ldots,\tilde{H}_{l}^{n} (\Im )} \right) \le DPLDPWGA\left( {\tilde{H}{^{\prime}_{l}}^{1} (\Im )\,,\tilde{H}{^{\prime}_{l}}^{2} (\Im )\,,\ldots,\tilde{H}{^{\prime}_{l}}^{n} (\Im )} \right).$

### Theorem 3.10 (Boundedness)

If $$\tilde{H}_{l}^{m - } (\Im ) = \left\langle {\{ \mathop {\min }\limits_{c} l_{{\varphi^{m(c)} }} (\Im^{m(c)} )\} ,\{ \mathop {\max }\limits_{d} l_{{\eta^{m(d)} }} (\Im^{m(d)} )\} } \right\rangle \,$$ and $$\tilde{H}_{l}^{m + } (\Im ) = \left\langle {\{ \mathop {\max }\limits_{c} l_{{\varphi^{m(c)} }} (\Im^{m(c)} )\} ,\{ \mathop {\min }\limits_{d} l_{{\eta^{m(d)} }} (\Im^{m(d)} )\} } \right\rangle \,$$ then $$\tilde{H}_{l}^{m - } \left( \Im \right) \prec DPLDPWGA\left( {\tilde{H}_{l}^{1} (\Im )\,,\tilde{H}_{l}^{2} (\Im )\,,\ldots,\tilde{H}_{l}^{n} (\Im )} \right)$$$$\prec \tilde{H}_{l}^{m + } \left( \Im \right).$$

## DPL-FUCOM-ARAS Method

**Fig. 2:**
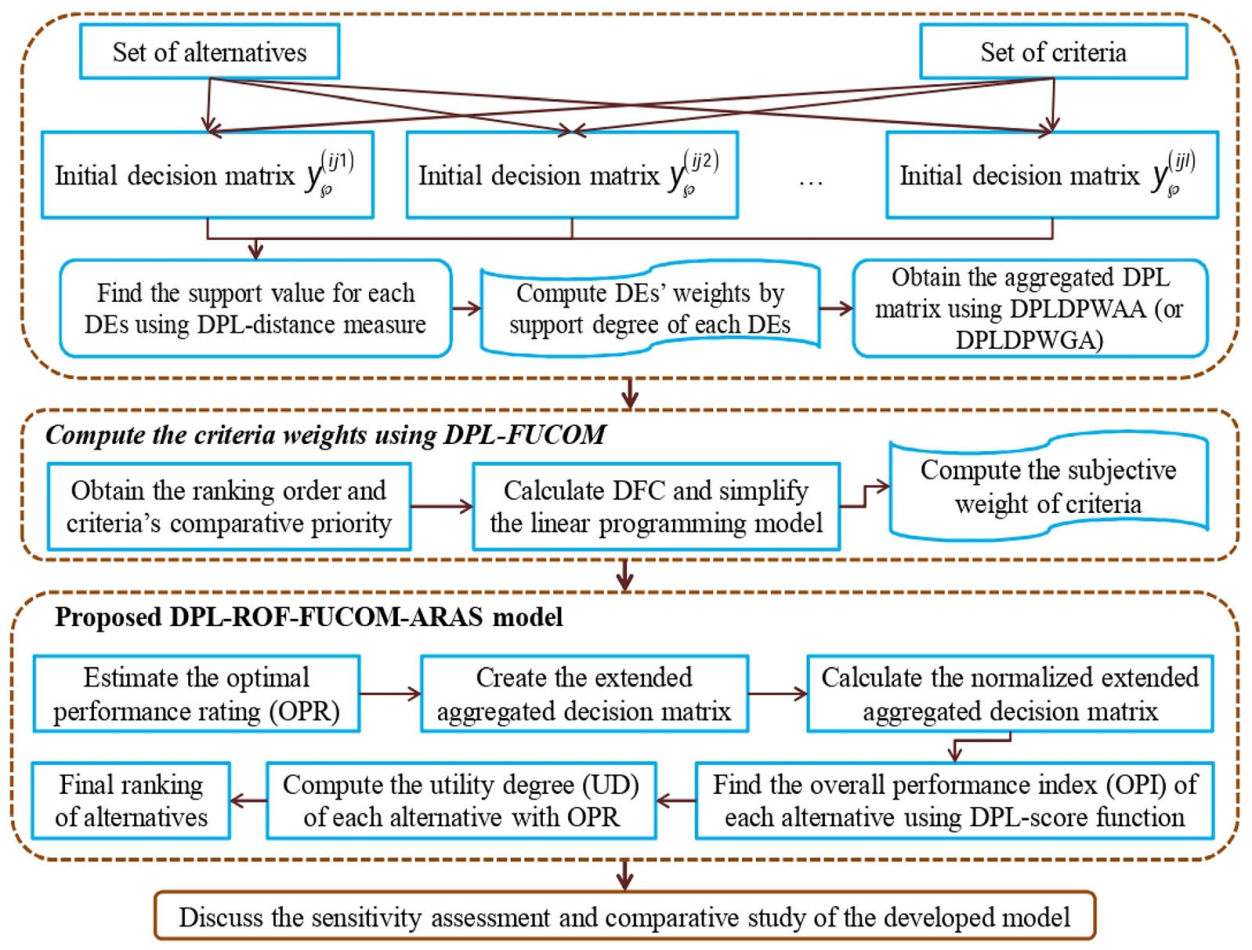
The representation of the DPL-FUCOM-ARAS method

Below is a description of the DPL-FUCOM-ARAS method’s operational procedures (see Fig. 2).

*Step 1* Suppose that under *n* different attributes $C_{j\,} \,(j = 1,2,\ldots,n)$, *m* different options $A_{i\,} \,(i = 1,2,\ldots,m)$ are to be assessed by the experts $D_{k\,} \,(k = 1,2,\ldots,r)$ with DPL data. Assume that the initial assessment of the DME *D*_*k*_ is represented by $\Re_{k} = \left[ {H_{l}^{ijk} \left( \Im \right)} \right]_{m \times n} = \left[ {\left\langle {\{ l_{{\varphi^{ijk(c)} }} (\Im^{(c)} )\} ,\{ l_{{\eta^{ijk(d)} }} (\Im^{(d)} )\} } \right\rangle } \right]_{m \times n} .$ Suppose, the given LTS is $T = \left\{ {l_{\beta } :\beta = - k,\ldots,k} \right\}.$

*Step 2* Obtain the DMEs’ initial assessment results expressed as adjusted DPLEs $\tilde{H}_{l}^{ijk} (\Im )$.

*Step 3* Compute the supports $SP\left( {\tilde{H}_{l}^{ijk} (\Im ),\tilde{H}_{l}^{ijt} (\Im )} \right),\,k \ne t$ and is shown as

14 $$SP\left( {\tilde{H}_{l}^{ijk} (\Im ),\tilde{H}_{l}^{ijt} (\Im )} \right) = 1 - D\left( {\tilde{H}_{l}^{ijk} (\Im ),\tilde{H}_{l}^{ijt} (\Im )} \right),\,\,k \ne t,$$

where $D\left( {\tilde{H}_{l}^{ijk} (\Im ),\tilde{H}_{l}^{ijt} (\Im )} \right)\,$ is distance between DPLEs $\tilde{H}_{l}^{ijk} (\Im )\,\,\& \,\,\tilde{H}_{l}^{ijt} (\Im )\,$ as follows:

15 $$D(\tilde{H}_{l}^{ijk} (\Im ),\tilde{H}_{l}^{ijt} (\Im ))\, = \sqrt {0.5\left( {\sum\limits_{c}^{{}} {\left( {\Im^{(c)} \times \left| {L(l_{{\varphi^{ijk(c)} }} ) - L(l_{{\varphi^{ijt(c)} }} )} \right|} \right)} + \sum\limits_{d}^{{}} {\left( {\Im^{(d)} \times \left| {L(l_{{\eta^{ijk(d)} }} ) - L(l_{{\eta^{ijt(d)} }} )} \right|} \right)} } \right)} .$$

*Step 4* Calculate the values $\varpi_{ijk}^{{}}$ utilizing Eq. (15).

16 $$\varpi_{ijk}^{{}} = \frac{{\delta_{k} \left( {1 + \sum\limits_{t = 1,k \ne t}^{r} {SP(\tilde{H}_{l}^{ijk} (\Im ),\tilde{H}_{l}^{ijt} (\Im ))} } \right)}}{{\sum\nolimits_{k = 1}^{r} {\delta_{k} \left( {1 + \sum\limits_{t = 1,k \ne t}^{r} {SP(\tilde{H}_{l}^{ijk} (\Im ),\tilde{H}_{l}^{ijt} (\Im ))} } \right)} }}\,\,\,$$

where $\delta_{k}$ is weight of the DME $D_{k}$ and clearly,$\sum\limits_{k = 1}^{r} {\varpi_{ijk}^{{}} = } 1$.

*Step 5* Aggregate the individual matrices.

Utilize *DPLDPWAA* or *DPLDPWGA* operator to generate the aggregated dual probabilistic linguistic decision matrix (ADPLDM)$\left[ {\tilde{H}_{l}^{ij} \left( \Im \right)} \right]_{m \times n} = \left[ {\left\langle {\{ l_{{\varphi^{ij(c)} }} (\Im^{(c)} )\} ,\{ l_{{\eta^{ij(d)} }} (\Im^{(d)} )\} } \right\rangle } \right]_{m \times n} .$

17 $$\begin{aligned} & \tilde{H}_{l}^{ij} (\Im ) = DPLDPWAA\left( {\tilde{H}_{l}^{ij1} (\Im ),\tilde{H}_{l}^{ij2} (\Im ),\ldots,\tilde{H}_{l}^{ijr} (\Im )} \right) \hfill \\ & \qquad\quad = \left\langle {\left\{ {L^{ - 1} \left( {1 - \left( {1 + \left\{ {\sum\limits_{k = 1}^{r} {\varpi_{ijk} \left( {\frac{{L_{\varphi }^{ijk} }}{{1 - L_{\varphi }^{ijk} }}} \right)^{Q} } } \right\}^{\frac{1}{Q}} } \right)^{ - 1} } \right)\,(\Im^{(c)} )} \right\},\,\left\{ {L^{ - 1} \left( {\left( {1 + \left\{ {\sum\limits_{k = 1}^{r} {\varpi_{ijk} \left( {\frac{{1 - L_{\eta }^{ijk} }}{{L_{\eta }^{ijk} }}} \right)^{Q} } } \right\}^{\frac{1}{Q}} } \right)^{ - 1} } \right)\,(\Im^{(d)} )} \right\}} \right\rangle \hfill \\ \end{aligned}$$

or

18 $$\begin{aligned} & \tilde{H}_{l}^{ij} (\Im ) = DPLDPWGA\left( {\tilde{H}_{l}^{ij1} (\Im ),\tilde{H}_{l}^{ij2} (\Im ),\ldots,\tilde{H}_{l}^{ijr} (\Im )} \right) \hfill \\ & \qquad\quad = \left\langle {\left\{ {L^{ - 1} \left( {\left( {1 + \left\{ {\sum\limits_{k = 1}^{r} {\varpi_{ijk} \left( {\frac{{1 - L_{\varphi }^{ijk} }}{{L_{\varphi }^{ijk} }}} \right)^{Q} } } \right\}^{\frac{1}{Q}} } \right)^{ - 1} } \right)\,(\Im^{(c)} )} \right\},\,\left\{ {L^{ - 1} \left( {1 - \left( {1 + \left\{ {\sum\limits_{k = 1}^{r} {\varpi_{ijk} \left( {\frac{{L_{\eta }^{ijk} }}{{1 - L_{\eta }^{ijk} }}} \right)^{Q} } } \right\}^{\frac{1}{Q}} } \right)^{ - 1} } \right)\,(\Im^{(d)} )} \right\}} \right\rangle \hfill \\ \end{aligned}$$

*Step 6* Use FUCOM for estimation of attribute weights.

In the context of making decisions, the determination of criteria weights is seen as a real issue because it can border on subjectivity in some cases. This approach has been acquiring significant importance and affects the outcome of DM situations due to the significant influence that weight coefficients have on the solution in various methods. The criteria weights in this study are calculated using the FUCOM approach. The complete consistency method (FUCOM) is the best in accordance with doctrine of comparisons in pairs of characteristics and the validation of outcomes by expressing divergence from the utmost consistency [21]. This method has further utilized for various purposes [53–57]. Here, we present the FUCOM model under DPL settings.

The steps of FUCOM are as below:

*Step 6.1* This stage attempts to rank the assessment attributes *C*_1_, *C*_2_, …,*C*_*n*_ from the very beginning.

The importance of the qualities is used to define the priority order, which runs from the most considerable criteria to the least considerable criteria. As a result, the weights’ achieved desired values allow us to construct the criteria’s ranking, which is as follows:

19 $$C_{j\left( 1 \right)} > C_{j\left( 2 \right)} > C_{j\left( 3 \right)} > \cdots > C_{j\left( \sigma \right)} ,$$

where $\mu_{ijk}$ denotes the observed criterion’s rank [21].

*Step 6.2* This stage aims to carry out a comparative analysis of ranked criteria as well as $\left( {\eta_{{{\sigma \mathord{\left/ {\vphantom {\sigma {\left( {\sigma + 1} \right)}}} \right. \kern-0pt} {\left( {\sigma + 1} \right)}}}} :\,\sigma \in \Lambda_{n} } \right)$ to establish the relative importance of the evaluation criteria. Importantly, when compared to $C_{{j\left( {\sigma + 1} \right)}}$ that of the $C_{j\left( \sigma \right)} ,$ the relative priority $\eta_{{{\sigma \mathord{\left/ {\vphantom {\sigma {\left( {\sigma + 1} \right)}}} \right. \kern-0pt} {\left( {\sigma + 1} \right)}}}}$ of the rank-related evaluation criterion is given preference. This leads to the following expression:

20 $$\psi = \left( {\eta_{{{1 \mathord{\left/ {\vphantom {1 2}} \right. \kern-0pt} 2}}} ,\eta_{{{2 \mathord{\left/ {\vphantom {2 3}} \right. \kern-0pt} 3}}} ,\ldots,\eta_{{{\sigma \mathord{\left/ {\vphantom {\sigma {\left( {\sigma + 1} \right)}}} \right. \kern-0pt} {\left( {\sigma + 1} \right)}}}} } \right),$$

wherein significance is followed by $\eta_{{{\sigma \mathord{\left/ {\vphantom {\sigma {\left( {\sigma + 1} \right)}}} \right. \kern-0pt} {\left( {\sigma + 1} \right)}}}}$, the rank of criterion $C_{j\left( \sigma \right)}$ being evaluated by rank of criterion $C_{{j\left( {\sigma + 1} \right)}} .$

*Step 6.3* The results of the weighted consideration $\left( {w_{1} ,w_{2} ,\ldots,w_{n} } \right)^{T}$ of criteria must be computed for this phase. The two restrictions that the final criteria weight results abide with are listed below.

- The criterion outlined below must be met, as the relative importance of the selected criteria is correlated with the ratio of weight coefficients:
  21 $$\frac{{w_{\sigma } }}{{w_{\sigma + 1} }} = \eta_{{{\sigma \mathord{\left/ {\vphantom {\sigma {\left( {\sigma + 1} \right)}}} \right. \kern-0pt} {\left( {\sigma + 1} \right)}}}} .$$
- The constraint $\eta_{{{\sigma \mathord{\left/ {\vphantom {\sigma {\left( {\sigma + 1} \right)}}} \right. \kern-0pt} {\left( {\sigma + 1} \right)}}}} \times \eta_{{{{\left( {\sigma + 1} \right)} \mathord{\left/ {\vphantom {{\left( {\sigma + 1} \right)} {\left( {\sigma + 2} \right)}}} \right. \kern-0pt} {\left( {\sigma + 2} \right)}}}} = \eta_{{{\sigma \mathord{\left/ {\vphantom {\sigma {\left( {\sigma + 2} \right)}}} \right. \kern-0pt} {\left( {\sigma + 2} \right)}}}}$ should be satisfied by weights of attributes. Since $\frac{{w_{\sigma } }}{{w_{\sigma + 1} }} = \eta_{{{\sigma \mathord{\left/ {\vphantom {\sigma {\left( {\sigma + 1} \right)}}} \right. \kern-0pt} {\left( {\sigma + 1} \right)}}}}$ and $\frac{{w_{\sigma + 1} }}{{w_{\sigma + 2} }} = \eta_{{{{\left( {\sigma + 1} \right)} \mathord{\left/ {\vphantom {{\left( {\sigma + 1} \right)} {\left( {\sigma + 2} \right)}}} \right. \kern-0pt} {\left( {\sigma + 2} \right)}}}}$, so $\frac{{w_{\sigma } }}{{w_{\sigma + 2} }} = \frac{{w_{\sigma } }}{{w_{\sigma + 1} }} \times \frac{{w_{\sigma + 1} }}{{w_{\sigma + 2} }}$; so, for establishing the final ratings of weights of considered characteristics, an additional constraint must be defined. This restriction’s definition is as follows:
  22 $$\frac{{w_{\sigma } }}{{w_{\sigma + 2} }} = \eta_{{{\sigma \mathord{\left/ {\vphantom {\sigma {\left( {\sigma + 1} \right)}}} \right. \kern-0pt} {\left( {\sigma + 1} \right)}}}} \times \eta_{{{{\left( {\sigma + 1} \right)} \mathord{\left/ {\vphantom {{\left( {\sigma + 1} \right)} {\left( {\sigma + 2} \right)}}} \right. \kern-0pt} {\left( {\sigma + 2} \right)}}}} .$$

It is crucial to note that successful transitivity is a requirement for the smallest deviation from “full consistency (DFC)” ($\Omega$), i.e., only when $\frac{{w_{\sigma } }}{{w_{\sigma + 1} }} = \eta_{{{\sigma \mathord{\left/ {\vphantom {\sigma {\left( {\sigma + 1} \right)}}} \right. \kern-0pt} {\left( {\sigma + 1} \right)}}}}$ and $\frac{{w_{\sigma } }}{{w_{\sigma + 2} }} = \eta_{{{\sigma \mathord{\left/ {\vphantom {\sigma {\left( {\sigma + 1} \right)}}} \right. \kern-0pt} {\left( {\sigma + 1} \right)}}}} \times \eta_{{{{\left( {\sigma + 1} \right)} \mathord{\left/ {\vphantom {{\left( {\sigma + 1} \right)} {\left( {\sigma + 2} \right)}}} \right. \kern-0pt} {\left( {\sigma + 2} \right)}}}}$ are considered. For implementation, the criteria weights must satisfy $\left| {\frac{{w_{\sigma } }}{{w_{\sigma + 1} }} - \eta_{{{\sigma \mathord{\left/ {\vphantom {\sigma {\left( {\sigma + 1} \right)}}} \right. \kern-0pt} {\left( {\sigma + 1} \right)}}}} } \right| \le \Omega$ and $\left| {\frac{{w_{\sigma } }}{{w_{\sigma + 2} }} - \eta_{{{\sigma \mathord{\left/ {\vphantom {\sigma {\left( {\sigma + 1} \right)}}} \right. \kern-0pt} {\left( {\sigma + 1} \right)}}}} \times \eta_{{{{\left( {\sigma + 1} \right)} \mathord{\left/ {\vphantom {{\left( {\sigma + 1} \right)} {\left( {\sigma + 2} \right)}}} \right. \kern-0pt} {\left( {\sigma + 2} \right)}}}} } \right| \le \Omega ,$ with the minimization of $\Omega .$ The following addresses the required method to achieve the final weights of considered criteria:

23 $$\left. \begin{gathered} \min \,\,\Omega \hfill \\ \left| {\frac{{w_{\sigma } }}{{w_{\sigma + 1} }} - \eta_{{{\sigma \mathord{\left/ {\vphantom {\sigma {\left( {\sigma + 1} \right)}}} \right. \kern-0pt} {\left( {\sigma + 1} \right)}}}} } \right| \le \Omega ,\,\forall \sigma \hfill \\ \left| {\frac{{w_{\sigma } }}{{w_{\sigma + 2} }} - \eta_{{{\sigma \mathord{\left/ {\vphantom {\sigma {\left( {\sigma + 1} \right)}}} \right. \kern-0pt} {\left( {\sigma + 1} \right)}}}} \times \eta_{{{{\left( {\sigma + 1} \right)} \mathord{\left/ {\vphantom {{\left( {\sigma + 1} \right)} {\left( {\sigma + 2} \right)}}} \right. \kern-0pt} {\left( {\sigma + 2} \right)}}}} } \right| \le \Omega ,\,\forall \sigma \hfill \\ w_{j} \ge 0,\,j = 1,2,\ldots,n\,\,{\text{with}}\,\,\sum\limits_{j = 1}^{n} {w_{j} = 1.} \hfill \\ \end{gathered} \right\}$$

Solution of (22) gives the final weights of criteria.

*Step 7* Create the extended ADPLM $\Re^{*}$.

Here, the extension is done based on optimal performance rating (OPR). Depending on profit and cost criteria, the OPR is given by

24 $$\tilde{H}_{l}^{{j + }} \left( \Im \right) = \left\{ {\begin{array}{ll} {\left\langle {\{ \mathop {\max }\limits_{i} \mathop {\max }\limits_{c} l_{{\varphi ^{{ij(c)}} }} (\mathop {\max }\limits_{c} \Im ^{{(c)}} )\} ,\{ \mathop {\min }\limits_{i} \mathop {\min }\limits_{d} l_{{\eta ^{{ij(d)}} }} (\mathop {\min }\limits_{d} \Im ^{{(d)}} )\} } \right\rangle ,{\mkern 1mu} \;{\text{for}}{\mkern 1mu} {\mkern 1mu} C_{j} \in Q_{B} } \\ {\left\langle {\{ \mathop {\min }\limits_{i} \mathop {\min }\limits_{d} l_{{\eta ^{{ij(d)}} }} (\mathop {\min }\limits_{d} \Im ^{{(d)}} )\} ,\{ \mathop {\max }\limits_{i} \mathop {\max }\limits_{c} l_{{\varphi ^{{ij(c)}} }} (\mathop {\max }\limits_{c} \Im ^{{(c)}} )\} } \right\rangle ,\;{\text{for}}{\mkern 1mu} {\mkern 1mu} {\mkern 1mu} C_{j} \in Q_{C} ,} \\ \end{array} } \right.{\text{ }}$$

where $Q_{B} ,Q_{C}$ being the set of all beneficial and non-beneficial attributes, respectively.

The extended ADPLM $\Re^{*}$ is given by

$$\begin{gathered} \begin{array}{*{20}c} {\,\,\,\,\,\,\,\,\,\,\,\,\,\,\,\,\,\,C_{1} } & {\,\,\,\,\,\,\,\,\,\,\,\,\,C_{2} } & {\,\,\,\,\, \cdots } & {\,\,\,\,\,C_{n} } \\ \end{array} \hfill \\ \,\begin{array}{*{20}c} {A_{1} } \\ {A_{2} } \\ \vdots \\ {A_{m} } \\ {OPR} \\ \end{array} \left( {\begin{array}{*{20}c} {\tilde{H}_{l}^{11} (\Im )\,} & {\tilde{H}_{l}^{12} (\Im )} & \cdots & {\tilde{H}_{l}^{1n} (\Im )} \\ {\tilde{H}_{l}^{21} (\Im )} & {\tilde{H}_{l}^{22} (\Im )} & \cdots & {\tilde{H}_{l}^{2n} (\Im )} \\ \vdots & \vdots & \vdots & \vdots \\ {\tilde{H}_{l}^{m1} (\Im )} & {\tilde{H}_{l}^{m2} (\Im )} & \cdots & {\tilde{H}_{l}^{mn} (\Im )} \\ {\tilde{H}_{l}^{1 + } (\Im )} & {\tilde{H}_{l}^{2 + } (\Im )} & \cdots & {\tilde{H}_{l}^{n + } (\Im )} \\ \end{array} } \right) \hfill \\ \end{gathered}$$

*Step 8* Obtain the normalized extended ADPLM (NE-ADPLM) $\Re^{*N} = \left[ {\hat{H}_{l}^{ij} (\Im )} \right]_{m \times n} = \left[ { < \{ \hat{l}_{{\varphi^{ij(c)} }} (\Im^{(c)} )\} ,\{ \hat{l}_{{\eta^{ij(d)} }} (\Im^{(d)} )\} > } \right]_{m \times n},$ where

25 $$\hat{H}_{l}^{ij} (\Im ) = \left\{ {\begin{array}{*{20}c} {\left\langle {\left\{ {l_{{L_{\varphi }^{ij} /L_{\varphi }^{j + } }} (\Im^{(c)} )} \right\},\,\left\{ {l_{{L_{\eta }^{ij} /L_{\eta }^{j + } }} (\Im^{(d)} )} \right\}} \right\rangle ,\,\,\,\,\,\,\,\,\,\,{\text{if}}\,\,C_{j} \in Q_{B} } \\ {\left\langle {\left\{ {l_{{L_{\varphi }^{j + } /L_{\varphi }^{ij} }} (\Im^{(c)} )} \right\},\,\left\{ {l_{{L_{\eta }^{j + } /L_{\eta }^{ij} }} (\Im^{(d)} )} \right\}} \right\rangle ,\,\,\,\,\,\,\,\,\,\,\,{\text{if}}\,\,\,C_{j} \in Q_{C} } \\ \end{array} } \right.\,\,\,\,\,\,\,$$

where *Q*_*B*_ and *Q*_*B*_ represent the benefit-type and cost-type attributes, respectively.

*Step 9* Establish the weighted NE-ADPLM $\Re^{*NW} = \left[ {\hat{H}{^{\prime}_{l}}^{ij} (\Im )} \right]_{m \times n} = \left[ { < \{ \hat{l}^{\prime}_{{\varphi^{ij(c)} }} (\Im^{(c)} )\} ,\{ \hat{l}^{\prime}_{{\eta^{ij(d)} }} (\Im^{(d)} )\} > } \right]_{m \times n}$ where

26 $$\hat{H}{^{\prime}_{l}}^{ij} (\Im ) = \left\langle {l_{{w_{j} \times L(\hat{l}_{{\varphi^{ij(c)} }} )}} (\Im^{(c)} )\} ,\{ l_{{w_{j} \times L(\hat{l}_{{\eta^{ij(d)} }} )}} (\Im^{(d)} )} \right\rangle .$$

*Step 10* Find the overall performance index (OPI) of $A_{i} \,(i = 1,2, \ldots ,n)$.

With the use of Eq. (26) and weighted NE-ADPLM $\Re^{*NW} ,$ the OPI of each option is obtained as

27 $$N_{i} = \sum\limits_{j = 1}^{n} {S\left( {\hat{H}{^{\prime}_{l}}^{ij} (\Im )} \right)} ,\,\,i = 1,2, \ldots ,n.$$

*Step 11* Evaluate the “utility degree (UD)” of each option.

The UD of each option is obtained using Eq. (27) as

28 $$UD_{i} = \frac{{N_{i} }}{{N_{0} }},\,\,i = 1,2, \ldots ,n,$$

where $N_{0}$ is score value of OPR, which is given in Eq. (24).

*Step 12* Prioritize the options $A_{i} \,(i = 1,2,\ldots,n)$ and find the most suitable one.

Alternatives are ranked according to how useful they are overall.

## Case Study: Medical Equipment Supplier (MES) Assessment

### Problem Description

With the increasing development of urban population, the number of “advanced medical equipment (AME)” in hospitals is increasing worldwide. Advanced medical equipment not only improve the medical diagnose levels but also provide an important part in evolving scientific research in hospitals. The selection of medical equipment depends of number of different criteria and uncertain information; therefore, it is important to develop an MCGDA for assessing the MES problem. In the literature, few authors have suggested some assessment models to deal with the MES problem. In a study, Bahadori et al. [58] assessed the barriers and drivers of MES evaluation problem and then rank the equipment through “Vise Kriterijumska Optimizacija I Kompromisno Resenje (VIKOR)” method. A study has provided to scrutinize the relationship between hospitals and their suppliers. These studies are not able to tackle with DPL data. For the first time, we apply the proposed DPL-FUCOM-ARAS approach for dealing with the MES selection problem from DPL perspective. For this purpose, a team of three DMEs is created to find the rank of MESs. After initial screening and literature review, we consider three suppliers as options *A*_1_, *A*_2_, and *A*_3_ and four criteria *C*_1_, *C*_2_, *C*_3_ and *C*_4_ as beneficial types. Table 1 presents the detail description of considered criteria for MES problem [58–60].

**Table 1:** The assessment criteria for MES selection

Criteria	Description
Package and transport quality (*C*_1_)	Considers the total package and transportation qualities in MESs assessment
Quality (*C*_2_)	The degree of medical consumer products provided by the supplier
Imbursement terms (*C*_3_)	MESs’ skill to submit with the hospital terms of compensation
Timeliness (*C*_4_)	Measures the delivery time and daily protection efficiency

### Solution

We use the created DPL-FUCOM-ARAS model for evaluating the considered MESs. The following sequential steps are involved in the introduced framework:

*Step 1* Consider the LTS $T = \{ l_{\alpha } :\alpha = - 3, - 2, - 1,0,1,2,3\},$ where $l_{ - 3}$ = extremely poor, $l_{ - 2}$ = poor, $l_{ - 1}$ = moderately poor, $l_{0}$ = fair, $l_{1}$ = good, $l_{2}$ = very good, and $l_{3}$ = excellent. The evaluation outcomes by experts are: $\Re_{k} = \left[ {H_{l}^{ijk} \left( \Im \right)} \right]_{3 \times 4} = \left[ {\left\langle {\{ l_{{\varphi^{ijk(c)} }} (\Im^{(c)} )\} ,\{ l_{{\eta^{ijk(d)} }} (\Im^{(d)} )\} } \right\rangle } \right]_{3 \times 4} ,k = 1,2,3$ (see Table 2).

**Table 2:** Initial assessment matrix

		C_1_	C_2_	C_3_	C_4_
D_1_	A_1_	< {*l*_-2_(0.3), *l*_-1_(0.4), *l*_1_(0.3)},{* l*_-1_(0.3), *l*_0_(0.7)} >	< {* l*_1_(0.6), *l*_2_(0.4)}, {* l*_-2_(0.6), *l*_1_(0.4)} >	< {* l*_-1_(0.6), *l*_1_(0.4)}, {* l*_0_(0.1), *l*_2_(0.9)} >	< {* l*_0_(1)}, {* l*_-2_(0.3), *l*_-1_(0.6), *l*_1_(0.1)} >
	A_2_	< {* l*_-1_(0.1),* l*_0_(0.9)}, {* l*_-2_(0.6), *l*_-1_(0.3),*l*_1_(0.1)} >	< {* l*_-2_(0.7), *l*_1_(0.3)}, {* l*_1_(0.3), *l*_4_(0.6)} >	< {* l*_0_(0.2), *l*_2_(0.8)}, {* l*_-1_(0.8), *l*_1_(0.2)} >	< {* l*_-2_(0.2), *l*_-1_(0.3), *l*_1_(0.5)},{* l*_0_(1)} >
	A_3_	< {* l*_1_(1)}, {* l*_0_(0.1), *l*_2_(0.9)} >	< {* l*_-1_(0.3), *l*_0_(0.7)}, {* l*_-2_(0.5), *l*_-1_(0.3), *l*_1_(0.2)} >	< {* l*_-2_(0.8), *l*_-1_(0.2)}, {* l*_-1_(0.2), *l*_0_(0.8)} >	< {* l*_2_(1)}, {* l*_-2_(0.3), *l*_1_(0.7)} >
D_2_	A_1_	< {* l*_-1_(0.7),* l*_1_(0.3)}, {* l*_-2_(0.7), *l*_-1_(0.3)} >	< {* l*_-2_(0.6), *l*_-1_(0.4)}, {* l*_2_(1)} >	< {* l*_-2_(0.1), *l*_-1_(0.5), *l*_1_(0.4)},{* l*_1_(1)} >	< {* l*_1_(0.5), *l*_2_(0.5)}, {* l*_-1_(1)} >
	A_2_	< {* l*_0_(0.1), *l*_2_(0.9)}, {* l*_-1_(0.9), *l*_1_(0.1)} >	< {* l*_-2_(0.5), *l*_-1_(0.3), *l*_1_(0.2)},{* l*_0_(1)} >	< {* l*_-1_(0.2), *l*_0_(0.8)}, {* l*_-2_(0.1), *l*_-1_(0.7), *l*_1_(0.2)} >	< {* l*_-2_(0.3),* l*_1_(0.7)}, {* l*_1_(0.3), *l*_2_(0.7)} >
	A_3_	< {* l*_-2_(0.9),* l*_-1_(0.1)}, {* l*_-1_(0.8), *l*_1_(0.2)} >	< {* l*_2_(1)}, {* l*_-2_(0.7), *l*_-1_(0.3)} >	< {* l*_1_(1)},{* l*_-2_(0.2), *l*_-1_(0.7), *l*_1_(0.1)} >	< {* l*_-1_(0.3),* l*_0_(0.7)}, {* l*_1_(0.3), *l*_0_(0.7)} >
D_3_	A_1_	< {* l*_0_(0.3), *l*_2_(0.7)}, {* l*_-2_(0.4), *l*_-1_(0.6)} >	< {* l*_-2_(0.4), *l*_-1_(0.5), *l*_1_(0.1)},{* l*_2_(1)} >	< {* l*_-1_(0.1), *l*_1_(0.3), *l*_0_(0.6)},{* l*_1_(1)} >	< {* l*_-2_(0.5),* l*_1_(0.5)}, {* l*_-1_(0.6), *l*_0_(0.4)} >
	A_2_	< {* l*_-2_(0.8),* l*_-1_(0.2)}, {* l*_0_(0.6), *l*_2_(0.4)} >	< {* l*_2_(1)},{* l*_-2_(0.2), *l*_-1_(0.2), *l*_1_(0.6)} >	< {* l*_1_(1)}, {* l*_-1_(0.1),*l*_0_(0.9)} >	< {* l*_-1_(0.3),* l*_0_(0.7)}, {* l*_-2_(0.3), *l*_1_(0.7)} >
	A_3_	< {* l*_-1_(0.9),* l*_1_(0.1)}, {* l*_-1_(0.8),* l*_1_(0.2)} >	< {* l*_0_(1)},{* l*_-1_(1)} >	< {* l*_-2_(0.1), *l*_-1_(0.7), *l*_1_(0.2)},{* l*_-2_(0.2), *l*_-1_(0.7), *l*_1_(0.1)} >	< {* l*_1_(0.3), *l*_2_(0.7)}, {* l*_1_(1)} >

*Step 2* The DMEs provided the initial assessment results DMEs using DPLTSs. Additional results are given in Table S1 of supplementary file.

*Step 3* We calculate the supports $SP\left( {\tilde{H}_{l}^{ijk} (\Im ),\tilde{H}_{l}^{ijt} (\Im )} \right),\,k \ne t$ using Eqs. (14) and (15) and we signify as $S^{(kt)} \,(\,k \ne t)$ and are given in Table 3.

**Table 3:** Support degrees for each criterion

	C_1_	C_2_	C_3_	C_4_
$S^{(12)} \left( { = S^{(21)} } \right)$	0.6995	0.2988	0.6445	0.5654
	0.6218	0.5420	0.5575	0.5286
	0.3365	0.4664	0.4836	0.3888
$S^{(32)} \left( { = S^{(23)} } \right)$	0.5670	0.8419	0.6730	0.5590
	0.4275	0.3888	0.5718	0.6348
	0.7673	0.4986	0.6292	0.4769
$S^{(31)} \left( { = S^{(13)} } \right)$	0.4972	0.3169	0.5257	0.6163
	0.4877	0.3809	0.5670	0.5850
	0.3786	0.7261	0.6406	0.6838

*Step 4* Using Eq. (16), $\varpi_{ijk}^{{}}$ are obtained in Table 4 by taking $\delta_{1} = 0.32,\,\,\delta_{2} = 0.35,\delta_{3} = 0.33$.

**Table 4:** Values of $\varpi_{ijk}^{{}}$

D_1_	C_1_	C_2_	C_3_	C_4_
A_1_	0.33654	0.273144	0.324569	0.336605
A_2_	0.347298	0.341951	0.332332	0.325324
A_3_	0.28754	0.343534	0.32646	0.33982

D_2_	C_1_	C_2_	C_3_	C_4_
A_1_	0.347227	0.361902	0.346602	0.32777
A_2_	0.337392	0.343356	0.333089	0.332997
A_3_	0.352696	0.307885	0.324706	0.305904

D_3_	C_1_	C_2_	C_3_	C_4_
A_1_	0.316234	0.364955	0.32883	0.335626
A_2_	0.31531	0.314693	0.334578	0.341679
A_3_	0.359764	0.348581	0.348834	0.354275

*Step 5* The ADPLM (see Table 5) is formed by making use of Eq. (17) and taking $Q = 3$.

**Table 5:** Aggregated-DPLM for MES assessment

	C_1_	C_2_	C_3_	C_4_
A_1_	< {* l*_-0.502_(0.3), *l*_1.639_(0.2), *l*_1.639_(0.2), *l*_1.683_(0.3)}, {* l*_-1.890_(0.3), *l*_-1.882_(0.1), *l*_-1.692_(0.3), *l*_-0.842_(0.3)} >	< {* l*_0.389_(0.4), *l*_0.399_(0.2), *l*_1.587_(0.3), *l*_1.61_(0.1)}, {* l*_-1.586_(0.3), *l*_-1.586_(0.3), *l*_1.471_(0.1), *l*_1.471_(0.3)} >	< {* l*_-1.170_(0.1), *l*_0.494_(0.3), *l*_-0.439_(0.2), *l*_0.846_(0.4)}, {* l*_0.443_(0.1), *l*_1.157_(0.3), *l*_1.157_(0.3), *l*_1.157_(0.3)} >	< {* l*_0.536_(0.2), *l*_0.536_(0.3), *l*_1.675_(0.3), *l*_1.675_(0.2)}, {* l*_-1.700_(0.3), *l*_-1_(0.3), *l*_-0.842_(0.3), *l*_-0.543_(0.1)} >
A_2_	< {* l*_-0.475_(0.1), *l*_1.663_(0.5), *l*_1.663_(0.2), *l*_1.664_(0.2)}, {* l*_-1.694_(0.6), *l*_-0.828_(0.1), *l*_-0.828_(0.2), *l*_1.152_(0.1)} >	< {* l*_1.636_(0.5), *l*_1.637_(0.2), *l*_1.660_(0.1), *l*_1.681_(0.2)}, {* l*_-1.640_(0.2), *l*_-0.527_(0.1), *l*_-0.520_(0.1), *l*_0.472_(0.6)} >	< {* l*_0.551_(0.2), *l*_1.679_(0.2), *l*_1.679_(0.5), *l*_1.679_(0.1)}, {* l*_-1.697_(0.1), *l*_-0.843_(0.6), *l*_-0.843_(0.1), *l*_0.433_(0.2)} >	< {℘_-1.400_(0.2), *l*_-1.162_(0.1), *l*_0.550_(0.2), *l*_0.839_(0.5)}, {* l*_-1.668_(0.1), *l*_-1.668_(0.2), *l*_0.491_(0.6), *l*_0.491_(0.1)} >
A_3_	< {* l*_0.423_(0.6), *l*_0.423_(0.1), *l*_0.423_(0.2), *l*_0.806_(0.1)}, {* l*_-0.869_(0.1), *l*_-0.846_(0.5), *l*_-0.846_(0.2), *l*_1.136_(0.2)} >	< {* l*_1.632_(0.2), *l*_1.632_(0.1), *l*_1.635_(0.1), *l*_1.635_(0.6)}, {* l*_-1.885_(0.5), *l*_-1.675_(0.2), *l*_-1_(0.1), *l*_-0.812_(0.2)} >	< {* l*_0.474_(0.1), *l*_0.481_(0.6), *l*_0.481_(0.1), *l*_0.824_(0.2)}, {* l*_-1.894_(0.2), *l*_-0.847_(0.2), *l*_-0.847_(0.5), *l*_0.441_(0.1)} >	< {* l*_1.685_(0.1), *l*_1.685_(0.2), *l*_1.895_(0.6), *l*_1.895_(0.1)}, {* l*_-1.664_(0.2), *l*_-1.664_(0.1), *l*_0.463_(0.2), *l*_0.463_(0.5)} >

*Step 6* We obtain the attribute weights by FUCOM.

*Step 6.1* The priority order is as C_4_ > C_1_ > C_3_ > C_2_.

*Step 6.2* Based on the scale $\left[ {1,9} \right]$ and in relation to the top-ranked C_4_ criterion, a comparison was established (See Table 6).

**Table 6:** Priorities of attributes

Attribute	C_4_	C_1_	C_3_	C_2_
$\varpi_{{C_{j(k)} }}$	1	1.6	2.8	4.6

*Step 6.3* Corresponding to the determined prioritization of criteria, we present the analysis model as

$$\begin{gathered} \min \chi \hfill \\ s.t.\,\,\left\{ \begin{gathered} \left| {\frac{{w_{4} }}{{w_{1} }} - 1.6} \right| \le \chi , \, \left| {\frac{{w_{1} }}{{w_{3} }} - 1.75} \right| \le \chi , \, \left| {\frac{{w_{3} }}{{w_{2} }} - 1.64} \right| \le \chi , \, \hfill \\ \left| {\frac{{w_{4} }}{{w_{3} }} - 2.80} \right| \le \chi , \, \left| {\frac{{w_{1} }}{{w_{2} }} - 2.88} \right| \le \chi , \, \sum\limits_{j = 1}^{4} {w_{j} } = 1, \, w_{j} \ge 0,\forall j. \hfill \\ \end{gathered} \right. \hfill \\ \end{gathered}$$

The final weights and DFC of the findings are achieved by resolving the issue with Lingo 17.0 software, as discussed in Table 7.

**Table 7:** Criteria weight for MES assessment

Criteria	*w*_*1*_	*w*_*2*_	*w*_*3*_	*w*_*4*_
$w_{j}$	0.2844	0.0988	0.1623	0.4544

*Step 7* In accordance with Eq. (24), the OPR is obtained and mentioned in last row of Table S2 of Supplementary file. The extended ADPLM $\wp^{*}$ is given by Table S2 in Supplementary file.

*Step 8* Using Eq. (25) the normalized extended ADPLM is made (Table S3 in Supplementary file).

*Step 9* The weighted NE-ADPLM is constructed using Eq. (26) and presented in Table S4 of Supplementary file.

*Steps 10–11* The OPI of an alternative in relation to OPR is calculated using Eq. (27). The UD in relation to the OPR is determined through Eq. (28) (see Table 8).

**Table 8:** Utility values of MESs

	C_1_	C_2_	C_3_	C_4_	OPI	UD	Ranking position
A_1_	0.14184	0.02348	− 0.04568	0.13614	0.2557	0.4498	1
A_2_	0.14349	0.03509	0.06364	− 0.07924	0.1630	0.2866	3
A_3_	0.03575	0.06554	0.04222	0.08088	0.2244	0.3946	2
OPR	0.16304	0.05682	0.09306	0.25573	0.56865	–	

*Step 12* Based on the utility value, rank the MESs as $A_{1} \succ A_{3} \succ A_{2}$ and thus, $A_{1}$ is an appropriate choice among others.

## Discussion

The current part of this study is broken down into two parts: (1) we analyze the effect of the parameter Q and (2) a comparison of the created MCGDA methodology with the relevant current methodologies. Below, we cover the specifics of these two sub-sections.

### Effect of the Parameter ‘Q’

**Fig. 3:**
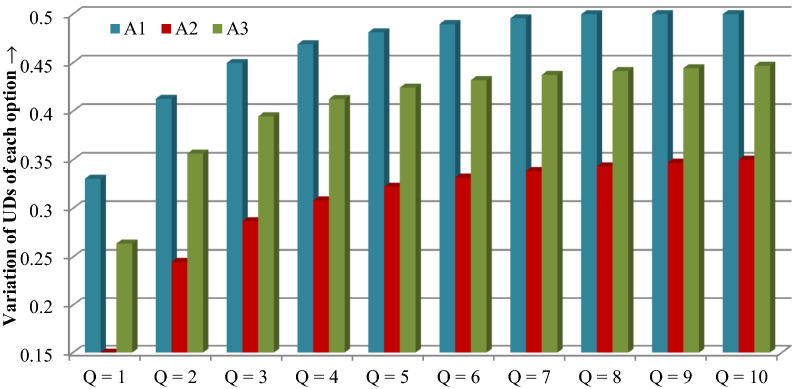
UDs of alternatives for different* Q*

We use the operator DPLDPWAA for various values of *Q* that fall within the range [1, 10] for investigating the effects of parameter ‘*Q*’ on final result. The utility values corresponding to diverse values of ‘*Q*’ are shown in Fig. 3.

From Fig. 3, we can observe that the total UDs that are derived from the DPLDPWAA operator are growing, with the rise in *Q* falling in the range of [1, 10]. Table 9 displays their matching ranking positions. According to different values of ‘*Q*’, we obtain that MES A_1_ has highest preference in all cases, A_2_ is ranked third in every instance, and A_3_ is ranked second in every instance. These findings indicate that, in comparison to all other alternatives, alternative A_1_ is more palatable. The “Spearman rank correlation coefficient (SRCC)” for various values of *Q* in [1, 10] that are invoked in Table 9 were then calculated [61]. Table 9 shows that the SRCC is 1, indicating the validity and dependability of our suggested methodology. In other words, during our designed procedure, the parameter *Q* did not exhibit any sensitive nature.

**Table 9:** Prioritization of MESs with respect to diverse values of ‘*Q*’

Options	*Q* = 1	*Q* = 2	*Q* = 3	*Q* = 4	*Q* = 5	*Q* = 6	*Q* = 7	*Q* = 8	*Q* = 9	*Q* = 10
A_1_	1	1	1	1	1	1	1	1	1	1
A_2_	3	3	3	3	3	3	3	3	3	3
A_3_	2	2	2	2	2	2	2	2	2	2
SRCC	1	1	1	1	1	1	1	1	1	1

### Comparative Study

This part presented an exploration into comparisons from both theoretical and numerical angles. We compare the proposed approach with some of the extant approaches, notably Xie et al. [16], Xie et al. [17], and Saha et al. [19] methods, which have good results in DPL settings. We implement the methods given by Xie et al. [16], Xie et al. [17] and Saha et al. [19] on the aforesaid case study that was previously described to demonstrate the efficacy of the created technique. Table 10 provides a summary of the findings.

**Table 10:** Comparison of proposed model with extant tools

Dynamics	Saha et al. [19]	Proposed	Xie et al. [16]	Xie et al. [17]
Type of data	DPLTS	DPLTS	DPLTS	DPLTS
Whether individual/group decision-making (GDM)?	GDM	GDM	GDM	GDM
Usage of probabilistic information	Yes	Yes	Yes	Yes
Adjustment of probabilities	Considered	Considered	No	No
Whether captures hesitation in preferences	No	Yes	Yes	Yes
If lessens the effects of reviewing information that is outrageously biased from some experts	Yes	Yes	No	No
Estimation of attributes weights	Criteria importance through inter-criteria correlation criteria (CRITIC)	FUCOM	Direct	Optimization technique
Relationship among attributes	Obtained	Computed (C_4_ > C_1_ > C_3_ > C_2_)	Not considered	Not considered
Operator(s) used	Generalized Dombi operator	Combination of PAO and Dombi operator	AOs	Preference relations
Operator’s flexibility	High	Very high	Very low	NA
Applied tool	MARCOS	ARAS	Nil	Nil
Sensitivity	Investigated	Investigated	Not investigated	Not investigated
Priority order	$\,A_{1} \succ A_{3} \succ A_{2}$	$\,A_{1} \succ A_{3} \succ A_{2}$	$\,A_{1} \succ A_{3} \succ A_{2}$	$\,A_{1} \succ A_{2} \succ A_{3}$

Table 10 outlines the benefits of our suggested approach in detail. The following can be deduced from analysis:

1. To manage uncertain data representation, we presented the dual probabilistic linguistic information-based decision support system, which actually increases the consistency and flexibility of the standard MCDA approaches.
2. The “reversible transformation functions (RTFs)” serve as the foundation for the suggested AOs. RFT’s ability to be used to convey the semantics of RFT, which give the semantic to LTs on various settings, is one of its advantages.
3. We have employed the idea of adjusted DPLEs to prevent situations when the output in the aggregate process is inappropriate.
4. Because Dombi operators have a parameter ‘Q’ with values that can be selected in accordance with actual decision needs, the proposed Dombi Power weighted AOs can more successfully aggregate the dual probabilistic linguistic information. Consequently, the methodology we discuss in this study can be considered one of the most useful tools created to date for tackling MCGDA challenges in a DPL setting.
5. To establish the weights of the criterion, the suggested method employed the FUCOM technique. FUCOM exhibited lesser departures of the realized degrees of the criterion from the most desirable values than other subjective weight-determining tools (the AHP, the BWM, and others) [21]. As a result, our approach lessens MCGDA process errors.

## Conclusions

As a generalized form of PLTSs, DPLTSs have been taken into consideration in this study to address the uncertainty and imprecision related to MCGDA problems. The arithmetic AOs have several limitations and lack of flexible parameters. In this context, some innovative operational laws for DPLTSs using Dombi operations have been proposed. Additionally, utilizing the idea of PAOs, we have proposed two aggregation operators as the DPLDPWAA and DPLDPWGA operators. We then have examined some of the generated AOs’ key properties, including idempotency, boundedness, monotonicity, etc. The most favorable alternative in a DPL setting is then obtained using an integrated DPL-FUCOM-ARAS-based MCGDA model based on the proposed AOs. In the DPL-FUCOM-ARAS model, the DPL-FUCOM model is used to calculate the criteria weights, and the DPL-ARAS model is proposed to rank the alternative. We have taken a case study of selecting of MES to further explain the practicality of the proposed DPL-FUCOM-ARAS methodology. By considering the sensitivity of the weighted criteria, we have demonstrated the feasibility of the proposed DPL-FUCOM-ARAS approach. We observe that the presented DPL-FUCOM-ARAS framework can be applied successfully in MCGDA problems in a DPLTS setting.

These findings have managerial implications that include as follows:

- The proposed DPL-FUCOM-ARAS methodology complements the DPL theory by offering a framework for decision-making that combines imprecise judgments that are essential to the selection of medical equipment suppliers.
- By suggesting novel operational rules, aggregation operators, and analytically realizing their essential properties, this study strengthens the theoretical foundation of DPLTSs.
- The PA operation combined with the Dombi operations decreases the impact of DME prioritizations and the methodical computation of weights of DMEs and criteria lessens errors and partialities in the MCGDA process with multiple experts.
- The sensitivity analysis and comparison inquiry demonstrate that the suggested method’s preference ordering for the alternatives are consistent with those of the other MCGDA strategies now in use. As a result, the suggested format is useful for capturing decision-makers’ opinions on the subject of choosing a medical equipment provider.

In the future, new MCGDA models with diverse integrated tools can be established for determining a practical solution to decision analysis problems, such as selection of “electric vehicle charging station (EVCS)” location, the treatment technology for medical waste, the choice of a technological forecasting method, the problem of choosing a cloud vendor, etc. Additionally, it is possible to build information measures for DPLTSs to determine the weights of the DMEs.

## Supplementary Information

Below is the link to the electronic supplementary material.

Supplementary file1 (DOCX 38 KB)
