# Supplementary material for: Dual Probabilistic Linguistic Full Consistency Additive Ratio Assessment Model for Medical Equipment Supplier Selection
Source: Int. J. Fuzzy Syst. 2023 May 8:1–17. Online ahead of print. doi: 10.1007/s40815-023-01526-w (PMC10166057; doi:10.1007/s40815-023-01526-w)
Supplement: Supplementary file 1 — Supplementary file1 (DOCX 38 KB) [file 40815_2023_1526_MOESM1_ESM.docx]

**Supplementary materials**

**Table S1:** Adjusted Initial decision matrix

|  |  | C_1_ | C_2_ | C_3_ | C_4_ |
| --- | --- | --- | --- | --- | --- |
| D_1_ | A_1_ | <{ *l* _-2_(0.3), *l* _-1_(0.2), *l* _-1_ (0.2), *l* _1_(0.3)},  { *l* _-1_(0.3), *l* _0_(0.1), *l* _0_ (0.3), *l* _0_(0.3)}> | <{ *l* _1_(0.4), *l* _1_(0.2), *l* _2_(0.3), *l* _2_(0.1)},  { *l* _-2_(0.3), *l* _-2_(0.3), *l* _1_(0.1), *l* _1_(0.3)}> | <{ *l* _-1_(0.1), *l* _-1_(0.3), *l* _-1_ (0.2), *l* _1_(0.4)},  { *l* _0_(0.1), *l* _2_(0.3), *l* _2_(0.3), *l* _2_(0.3)}> | <{ *l* _0_(0.2), *l* _0_(0.3), *l*_0_ (0.3), *l* _0_(0.2)},  { *l* _-2_(0.3), *l* _-1_(0.3), *l* _-1_ (0.3), *l* _1_(0.1)}> |
|  | A_2_ | <{ *l* _-1_(0.1), *l* _0_(0.5), *l*_0_ (0.2), *l* _0_(0.2)},  { *l* _-2_(0.6), *l* _-1_(0.1), *l* _-1_(0.2), *l* _1_(0.1)}> | <{ *l* _-2_(0.5), *l* _-2_(0.2), *l*_1_ (0.1), *l* _1_(0.2)},  { *l* _1_(0.2), *l* _1_(0.1), *l* _2_(0.1), *l*_2_ (0.6)}> | <{ *l* _0_(0.2), *l* _2_(0.2), *l*_2_ (0.5), *l* _2_(0.1)},  { *l* _-1_(0.1), *l* _-1_(0.6), *l*_-1_ (0.1), *l* _1_(0.2)}> | <{ *l* _-2_(0.2), *l* _-1_(0.1), *l*_-1_ (0.2), *l* _1_(0.5)},  { *l* _0_(0.1), *l* _0_(0.2), *l*_0_ (0.6), *l* _0_(0.1)}> |
|  | A_3_ | <{ *l* _1_(0.6), *l* _1_(0.1), *l*_1_ (0.2), *l* _1_(0.1)},  { *l* _0_(0.1), *l* _2_(0.5), *l*_2_ (0.2), *l* _2_(0.2)}> | <{ *l* _-1_(0.2), *l* _-1_(0.1), *l*_0_ (0.1), *l* _0_(0.6)},  { *l* _-2_(0.5), *l* _-1_(0.2),  *l* _-1_ (0.1), *l* _1_(0.2)}> | <{ *l* _-2_(0.1), *l* _-2_(0.6), *l*_-2_ (0.1), *l* _-1_(0.2)},  { *l* _-1_(0.2), *l* _0_(0.2), *l*_0_ (0.5), *l* _0_(0.1)}> | <{ *l* _2_(0.1), *l* _2_(0.2), *l*_2_ (0.6), *l* _2_(0.1)},  { *l* _-2_(0.2), *l* _-2_(0.1), *l*_1_ (0.2), *l* _1_(0.5)}> |
| D_2_ | A_1_ | <{ *l* _-1_(0.3), *l* _-1_(0.2), *l* _-1_ (0.2), *l* _1_(0.3)},  { *l* _-2_(0.3), *l* _-2_(0.1), *l* _-2_ (0.3), *l* _-1_(0.3)}> | <{ *l* _-2_(0.4), *l* _-2_(0.2), *l*_-1_ (0.3), *l* _-1_(0.1)},  { *l* _2_(0.3), *l* _2_(0.3), *l* _2_(0.1), *l*_2_ (0.3)}> | <{ *l* _-2_(0.1), *l* _-1_(0.3), *l*_-1_ (0.2), *l* _1_(0.4)},  { *l* _1_(0.1), *l* _1_(0.3), *l* _1_(0.3), *l* _1_(0.3)}> | <{ *l* _1_(0.2), *l* _1_(0.3), *l*_2_ (0.3), *l* _2_(0.2)},  { *l* _-1_(0.3), *l* _-1_(0.3), *l* _-1_ (0.3), *l* _-1_(0.1)}> |
|  | A_2_ | <{ *l* _0_(0.1), *l* _2_(0.5), *l*_2_ (0.2), *l* _2_(0.2)},  { *l* _-1_(0.6), *l* _-1_(0.1), *l* _-1_ (0.2), *l* _1_(0.1)}> | <{ *l* _-2_(0.5), *l* _-1_(0.2), *l* _-1_ (0.1), *l* _1_(0.2)},  { *l* _0_(0.2), *l* _0_(0.1), *l* _0_(0.1), *l*_0_ (0.6)}> | <{ *l* _-1_(0.2), *l* _0_(0.2), *l*_0_ (0.5), *l* _0_(0.1)},  { *l* _-2_(0.1), *l* _-1_(0.6), ℘_-1_ (0.1), *l* _1_(0.2)}> | <{ *l* _-2_(0.2), *l* _-2_(0.1), *l*_1_ (0.2), *l* _1_(0.5)},  { *l* _1_(0.1), *l* _1_(0.2), *l*_2_ (0.6), *l* _2_(0.1)}> |
|  | A_3_ | <{ *l* _-2_(0.6), *l* _-2_(0.1), *l* _-2_ (0.2), *l* _-1_(0.1)},  { *l* _-1_(0.1), *l* _-1_(0.5), *l* _-1_ (0.2), *l* _1_(0.2)}> | <{ *l* _2_(0.2), *l* _2_(0.1), *l* _2_(0.1), *l* _2_(0.6)},  { *l* _-2_(0.5), *l* _-2_(0.2),  *l* _-1_(0.1), *l* _-1_(0.2)}> | <{ *l* _1_(0.1), *l* _1_(0.6), *l*_1_ (0.1), *l* _1_(0.2)},  { *l* _-2_(0.2), *l* _-1_(0.2), *l* _-1_ (0.5), *l* _1_(0.1)}> | <{ *l* _-1_(0.1), *l* _-1_(0.2), *l* _0_ (0.6), *l* _0_(0.1)},  { *l* _1_(0.2), *l* _1_(0.1), *l* _0_ (0.2), *l* _0_(0.5)}> |
| D_3_ | A_1_ | <{ *l* _0_(0.3), *l* _2_(0.2), *l* _2_ (0.2), *l* _2_(0.3)},  { *l* _-2_(0.3), *l* _-2_(0.1), *l* _-1_ (0.3), *l* _-1_(0.3)}> | <{ *l* _-2_(0.4), *l* _-1_(0.2), *l* _-1_ (0.3), *l* _1_(0.1)},  { *l* _2_(0.3), *l* _2_(0.3), *l* _2_(0.1), *l*_2_ (0.3)}> | <{ *l* _-1_(0.1), *l* _1_(0.3), *l* _0_ (0.2), *l* _0_(0.4)},  { *l* _1_(0.1), *l* _1_(0.3), *l* _1_(0.3), *l* _1_(0.3)}> | <{ *l* _-2_(0.2), *l* _-2_(0.3), *l* _1_ (0.3), *l* _1_(0.2)},  { *l* _-1_(0.3), *l* _-1_(0.3), *l* _0_ (0.3), *l* _0_(0.1)}> |
|  | A_2_ | <{ *l* _-2_(0.1), *l* _-2_(0.5), *l* _-2_ (0.2), *l* _-1_(0.2)},  { *l* _0_(0.6), *l* _2_(0.1), *l* _2_ (0.2), *l* _2_(0.1)}> | <{ *l* _2_(0.5), *l* _2_(0.2), *l* _2_(0.1), *l* _2_(0.2)},  { *l* _-2_(0.2), *l* _-1_(0.1),  *l* _-1_(0.1), *l* _1_(0.6)}> | <{ *l* _1_(0.2), *l* _1_(0.2), *l* _1_ (0.5), *l* _1_(0.1)},  { *l* _-1_(0.1), *l* _0_(0.6), *l* _0_ (0.1), *l* _0_(0.2)}> | <{ *l* _-1_(0.2), *l* _-1_(0.1), *l* _0_ (0.2), *l* _0_(0.5)},  { *l* _-2_(0.1), *l* _-2_(0.2), *l* _1_ (0.6), *l* _1_(0.1)}> |
|  | A_3_ | <{ *l* _-1_(0.6), *l* _-1_(0.1), *l* _-1_ (0.2), *l* _1_(0.1)},  { *l* _-1_(0.1), *l* _-1_(0.5), *l* _-1_ (0.2), *l* _1_(0.2)}> | <{ *l* _0_(0.2), *l* _0_(0.1), *l* _0_(0.1), *l* _0_(0.6)},  { *l* _-1_(0.5), *l* _-1_(0.2),  *l* _-1_(0.1), *l* _-1_(0.2)}> | <{ *l* _-2_(0.1), *l* _-1_(0.6), *l* _-1_ (0.1), *l* _1_(0.2)},  { *l* _-2_(0.2), *l* _-1_(0.2), *l* _-1_ (0.5), *l* _1_(0.1)}> | <{ *l* _1_(0.1), *l* _1_(0.2), *l* _2_ (0.6), *l* _2_(0.1)},  { *l* _1_(0.2), *l* _1_(0.1), *l* _1_ (0.2), *l* _1_(0.5)}> |

**Table S2:** Extended Aggregated DPL matrix

|  | C_1_ | C_2_ | C_3_ | C_4_ |
| --- | --- | --- | --- | --- |
| A_1_ | <{ *l* _-0.502_(0.3), *l* _1.639_(0.2),  *l* _1.639_(0.2), *l* _1.683_(0.3)},  { *l* _-1.890_(0.3), *l* _-1.882_(0.1),  *l* _-1.692_(0.3), *l* _-0.842_(0.3)}> | <{ *l* _0.389_(0.4), *l* _0.399_(0.2),  *l* _1.587_(0.3), *l* _1.61_(0.1)},  { *l* _-1.586_(0.3), *l* _-1.586_(0.3),  *l* _1.471_(0.1), *l* _1.471_(0.3)}> | <{ *l* _-1.170_(0.1), *l* _0.494_(0.3), *l* _-0.439_(0.2), *l* _0.846_(0.4)},  { *l* _0.443_(0.1), *l* _1.157_(0.3),  *l* _1.157_(0.3), *l* _1.157_(0.3)}> | <{ *l* _0.536_(0.2), *l* _0.536_(0.3),  *l* _1.675_(0.3), *l* _1.675_(0.2)},  { *l* _-1.700_(0.3), *l* _-1_(0.3),  *l* _-0.842_(0.3), *l* _-0.543_(0.1)}> |
| A_2_ | <{ *l* _-0.475_(0.1), *l* _1.663_(0.5),  *l* _1.663_(0.2), *l* _1.664_(0.2)},  { *l* _-1.694_(0.6), *l* _-0.828_(0.1),  *l* _-0.828_(0.2), *l* _1.152_(0.1)}> | <{ *l* _1.636_(0.5), *l* _1.637_(0.2),  *l* _1.660_(0.1), *l* _1.681_(0.2)},  { *l* _-1.640_(0.2), *l* _-0.527_(0.1),  *l* _-0.520_(0.1), *l* _0.472_(0.6)}> | <{ *l* _0.551_(0.2), *l* _1.679_(0.2),  *l* _1.679_(0.5), *l* _1.679_(0.1)},  { *l* _-1.697_(0.1), *l* _-0.843_(0.6),  *l* _-0.843_(0.1), *l* _0.433_(0.2)}> | <{ *l* _-1.400_(0.2), ℘_-1.162_(0.1),  *l* _0.550_(0.2), *l* _0.839_(0.5)},  { *l* _-1.668_(0.1), *l* _-1.668_(0.2),  *l* _0.491_(0.6), *l* _0.491_(0.1)}> |
| A_3_ | <{ *l* _0.423_(0.6), *l* _0.423_(0.1),  *l* _0.423_(0.2), *l* _0.806_(0.1)},  { *l* _-0.869_(0.1), *l* _-0.846_(0.5),  *l* _-0.846_(0.2), *l* _1.136_(0.2)}> | <{ *l* _1.632_(0.2), *l* _1.632_(0.1),  *l* _1.635_(0.1), *l* _1.635_(0.6)},  { *l* _-1.885_(0.5), *l* _-1.675_(0.2),  *l* _-1_(0.1), *l* _-0.812_(0.2)}> | <{ *l* _0.474_(0.1), *l* _0.481_(0.6),  *l* _0.481_(0.1), *l* _0.824_(0.2)},  { *l* _-1.894_(0.2), *l* _-0.847_(0.2),  *l* _-0.847_(0.5), *l* _0.441_(0.1)}> | <{ *l* _1.685_(0.1), *l* _1.685_(0.2),  *l* _1.895_(0.6), *l* _1.895_(0.1)},  { *l* _-1.664_(0.2), *l* _-1.664_(0.1),  *l* _0.463_(0.2), *l* _0.463_(0.5)}> |
| OPR | <{ *l* _1.683_(0.6)},  { *l* _-1.890_(0.1)}> | <{ *l* _1.681_(0.6)},  { *l* _-1.885_(0.1)}> | <{ *l* _1.679_(0.6)},  { *l* _-1.894_(0.1)}> | <{ *l* _1.895_(0.6)},  { *l* _-1.700_(0.1)}> |

**Table S3:** Extended normalized Aggregated DPL matrix

|  | C_1_ | C_2_ | C_3_ | C_4_ |
| --- | --- | --- | --- | --- |
| A_1_ | <{ *l* _0.199_(0.3), *l* _2.943_(0.2),  *l* _2.943_(0.2), *l* _3_(0.3)},  { *l* _-1.396_(0.3), *l* _-1.384_(0.1),  *l* _-1.111_(0.3), *l* _0.118_(0.3)}> | <{ *l* _1.344_(0.4), *l* _1.356_(0.2),  *l* _2.878_(0.3), *l* _2.915_(0.1)},  { *l* _-1.103_(0.3), *l* _-1.103_(0.3),  *l* _3_(0.1), *l* _3_(0.3)}> | <{ *l* _-0.654_(0.1), *l* _1.480_(0.3), *l* _0.283_(0.2), *l* _1.931_(0.4)},  { *l* _1.969_(0.1), *l* _3_(0.3),  *l* _3_(0.3), *l* _3_(0.3)}> | <{ *l* _1.335_(0.2), *l* _1.335_(0.3),  *l* _2.730_(0.3), *l* _2.730_(0.2)},  { *l* _-0.767_(0.3), *l* _0.436_(0.3),  *l* _0.707_(0.3), *l* _1.221_(0.1)}> |
| A_2_ | <{ *l* _0.234_(0.1), *l* _2.975_(0.5),  *l* _2.975_(0.2), *l* _2.975_(0.2)},  { *l* _-1.112_(0.6), *l* _0.137_(0.1),  *l* _0.137_(0.2), *l* _3_(0.1)}> | <{ *l* _2.942_(0.5), *l* _2.942_(0.2),  *l* _2.972_(0.1), *l* _3_(0.2)},  { *l* _-1.175_(0.2), *l* _0.317_(0.1),  *l* _0.326_(0.1), *l* _1.659_(0.6)}> | <{ *l* _1.553_(0.2), *l* _3_(0.2),  *l* _3_(0.5), *l* _3_(0.1)},  { *l* _-1.120_(0.1), *l* _0.112_(0.6),  *l* _0.112_(0.1), *l* _1.954_(0.2)}> | <{ *l* _-1.040_(0.2), *l* _-0.747_(0.1),  *l* _1.351_(0.2), *l* _1.705_(0.5)},  { *l* _-0.711_(0.1), *l* _-0.711_(0.2),  *l* _3_(0.6), *l* _3_(0.1)}> |
| A_3_ | <{ *l* _1.386_(0.6), *l* _1.386_(0.1),  *l* _1.386_(0.2), *l* _1.876_(0.1)},  { *l* _0.078_(0.1), *l* _0.111_(0.5),  *l* _0.111_(0.2), *l* _2.977_(0.2)}> | <{ *l* _2.936_(0.2), *l* _2.936_(0.1),  *l* _2.940_(0.1), *l* _2.940_(0.6)},  { *l* _-1.504_(0.5), *l* _-1.223_(0.2),  *l* _-0.316_(0.1), *l* _-0.064_(0.2)}> | <{ *l* _1.454_(0.1), *l* _1.464_(0.6),  *l* _1.464_(0.1), *l* _1.902_(0.2)},  { *l* _-1.404_(0.2), *l* _0.105_(0.2),  *l* _0.105_(0.5), *l* _1.966_(0.1)}> | <{ *l* _2.743_(0.1), *l* _2.743_(0.2),  *l* _3_(0.6), *l* _3_(0.1)},  { *l* _-0.704_(0.2), *l* _-0.704_(0.1),  *l* _2.951_(0.2), *l* _2.951_(0.5)}> |
| OPR | <{ *l* _3_(0.6)},  { *l* _-1.396_(0.1)}> | <{ *l* _3_(0.6)},  { *l* _-1.504_(0.1)}> | <{ *l* _3_(0.6)},  { *l* _-1.404_(0.1)}> | <{ *l* _3_(0.6)},  { *l* _-0.767_(0.1)}> |

**Table S4:** Weighted Extended normalized Aggregated DPL matrix

|  | C_1_ | C_2_ | C_3_ | C_4_ |
| --- | --- | --- | --- | --- |
| A_1_ | <{ *l* _-2.090_(0.3), *l* _-1.309_(0.2),  *l* _-1.309_(0.2), *l* _-1.293_(0.3)},  { *l* _-2.544_(0.3), *l* _-2.540_(0.1),  *l* _-2.462_(0.3), *l* _-2.113_(0.3)}> | <{ *l* _-2.570_(0.4), *l* _-2.569_(0.2),  *l* _-2.419_(0.3), *l* _-2.415_(0.1)},  { *l* _-2.812_(0.3), *l* _-2.812_(0.3),  *l* _-2.407_(0.1), *l* _-2.407_(0.3)}> | <{ *l* _-2.619_(0.1), *l* _-2.272_(0.3), *l* _-2.467_(0.2), *l* _-2.199_(0.4)},  { *l* _-2.193_(0.1), *l* _-2.026_(0.3),  *l* _-2.026_(0.3), *l* _-2.026_(0.3)}> | <{ *l* _-1.030_(0.2), *l* _-1.030_(0.3),  *l* _-0.396_(0.3), *l* _-0.396_(0.2)},  { *l* _-1.985_(0.3), *l* _-1.438_(0.3),  *l* _-1.315_(0.3), *l* _-1.081_(0.1)}> |
| A_2_ | <{ *l* _-2.080_(0.1), *l* _-1.301_(0.5),  *l* _-1.301_(0.2), *l* _-1.301_(0.2)},  { *l* _-2.463_(0.6), *l* _-2.107_(0.1),  *l* _-2.107_(0.2), *l* _-1.293_(0.1)}> | <{ *l* _-2.412_(0.5), *l* _-2.412_(0.2),  *l* _-2.409_(0.1), *l* _-2.407_(0.2)},  { *l* _-2.819_(0.2), *l* _-2.672_(0.1),  *l* _-2.671_(0.1), *l* _-2.539_(0.6)}> | <{ *l* _-2.261_(0.2), *l* _-2.026_(0.2),  *l* _-2.026_(0.5), *l* _-2.026_(0.1)},  { *l* _-2.695_(0.1), *l* _-2.494_(0.6),  *l* _-2.494_(0.1), *l* _-2.195_(0.2)}> | <{ *l* _-2.109_(0.2), *l* _-1.976_(0.1),  *l* _-1.022_(0.2), *l* _-0.861_(0.5)},  { *l* _-1.960_(0.1), *l* _-1.960_(0.2),  *l* _-0.273_(0.6), *l* _-0.273_(0.1)}> |
| A_3_ | <{ *l* _-1.752_(0.6), *l* _-1.752_(0.1),  *l* _-1.752_(0.2), *l* _-1.613_(0.1)},  { *l* _-2.124_(0.1), *l* _-2.115_(0.5),  *l* _-2.115_(0.2), *l* _-1.299_(0.2)}> | <{ *l* _-2.413_(0.2), *l* _-2.413_(0.1),  *l* _-2.413_(0.1), *l* _-2.413_(0.6)},  { *l* _-2.852_(0.5), *l* _-2.824_(0.2),  *l* _-2.734_(0.1), *l* _-2.709_(0.2)}> | <{ *l* _-2.277_(0.1), *l* _-2.275_(0.6),  *l* _-2.275_(0.1), *l* _-2.204_(0.2)},  { *l* _-2.741_(0.2), *l* _-2.495_(0.2),  *l* _-2.495_(0.5), *l* _-2.193_(0.1)}> | <{ *l* _-0.390_(0.1), *l* _-0.390_(0.2),  *l* _-0.273_(0.6), *l* _-0.273_(0.1)},  { *l* _-1.956_(0.2), *l* _-1.956_(0.1),  *l* _-0.295_(0.2), *l* _-0.295_(0.5)}> |
| OPR | <{ *l* _-1.293_(0.6)},  { *l* _-2.544_(0.1)}> | <{ *l* _-2.407_(0.6)},  { *l* _-2.852_(0.1)}> | <{ *l* _-2.026_(0.6)},  { *l* _-2.741_(0.1)}> | <{ *l* _-0.273_(0.6)},  { *l* _-1.985_(0.1)}> |
